# Exploring the Potential Mechanism of Tang-Shen-Ning Decoction against Diabetic Nephropathy Based on the Combination of Network Pharmacology and Experimental Validation

**DOI:** 10.1155/2021/1025053

**Published:** 2021-09-09

**Authors:** Jiajun Liang, Jiaxin He, Yanbin Gao, Zhiyao Zhu

**Affiliations:** ^1^School of Traditional Chinese Medicine, Capital Medical University, Beijing 100069, China; ^2^Beijing Key Laboratory of TCM Collateral Disease Theory Research, Beijing 100069, China

## Abstract

**Background:**

Diabetic nephropathy (DN) has become one of the leading causes of the end-stage renal disease (ESRD). Tang-Shen-Ning (TSN) decoction, an effective Traditional Chinese formula for DN, can improve the renal function and inhibit renal fibrosis in DN. However, its potential mechanism is still unexplored.

**Methods:**

A network pharmacology approach was employed in this study, including screening for differential expressed genes of DN (DN-DEGs), protein-protein interaction (PPI) network analysis, and GO and KEGG enrichment analysis. Besides, a rat model was established to verify the potential effect of TSN in DN.

**Results:**

Twenty-three TSN-related DN-DEGs targets were identified. These genes were associated with decreased glomerular filtration rate (GFR) DN. The enrichment analysis suggested that the inhibition of renal fibrosis and inflammation through growth factors and chemokines is the potential mechanism through which TSN improves DN. TSN reduced renal fibrosis and improved pathological damage in the kidney in vivo through the regulation of GJA1, CTGF, MMP7, and CCL5, which are genes associated with ECM deposition.

**Conclusion:**

This study revealed that TSN improves DN through a multicomponent, multitarget, and multipathway synergy. We provide a scientific basis for potential targets for TSN use to treat DN, yet further experimental validation is needed to investigate these targets and mechanisms.

## 1. Introduction

Diabetic nephropathy (DN), a severe microvascular complication of long-term diabetes mellitus (DM), is present in 40% of diabetic patients [1]. DN leads to chronic progressive renal damage, accompanied by increased urinary proteins and decreased glomerular filtration rate (GFR). Indeed, DN becomes a leading cause of progression to end-stage renal disease (ESRD) in DM patients [2, 3]. KDOQI proposed that the term “diabetic nephropathy” should be replaced by “diabetic kidney disease” (DKD), which applies to a kidney disease that is caused by diabetes, while DN can be diagnosed only after the histopathological confirmation. The pathological progression of DN includes glomerular basement membrane thickening, mesangial matrix hyperplasia, and glomerulosclerosis [4]. DN's pathogenesis is complex and includes alterations in renal hemodynamics, disturbances in glucolipid metabolism, the action of various cytokines, and activation of the Renin-Angiotensin-Aldosterone System (RAAS). RAAS inhibitors have some benefit in the treatment of DN. However, according to clinical studies, when patients were treated with dual RAAS blocking, not only is it less effective in the long term, but patients were also more likely to suffer from risky events like acute kidney injury or hyperkalemia [5, 6]. Therefore, it is essential to identify novel therapeutic schedule for DN.

In China, Traditional Chinese Medicine (TCM) has a long history of treating DM and DN and provides renal protection for DN through different herbal combinations [7]. Developed by Yanbin Gao, a TCM expert, Tang-Shen-Ning decoction (TSN) is an empirical formula to treat DN, consisting of *Astragalus membranaceus* (Fisch.) Bunge (*Huang Qi* in Chinese, HQ, 15 g), *Euryale ferox Salisb.* ex DC (*Qian Shi* in Chinese, QS, 15 g), *Rosa laevigata* Michx (*Jin Ying Zi* in Chinese, JYZ, 15 g), *Rheum officinale* Baill (*Da Huang* in Chinese, DH, 6 g), and *Ligusticum chuanxiong* Hort (*Chuan Xiong* in Chinese, CX, 12 g). According to a double-blinded control clinical trial on DN patients, the use of TSN led to reductions in 24-hour urine albumin excretion rate (24 h UAER) compared to control group. Besides, patients in the TSN treatment group showed improvements in serum SOD, MDA, and hs-CRP compared to the control group, indicating that TSN is a safe and effective medicine for the treatment of early DN and improving the inflammatory and oxidative stress status in DN [8]. It was also shown that TSN treatment in DN mice decreased 24 h UAER, serum creatinine, and blood urea nitrogen. Furthermore, TSN activated the Wnt/*β*-catenin pathway, reversed the podocyte epithelial-mesenchymal transition (EMT), reduced the expression of fibroblast-specific protein 1 (FSP-1) and collagen I, and alleviated kidney damage in DN mice [9]. However, the effect of TCM is multitargeted and affects multiple pathways; therefore, the potential mechanism of TSN in DN still needs to be investigated.

**Figure 1:**
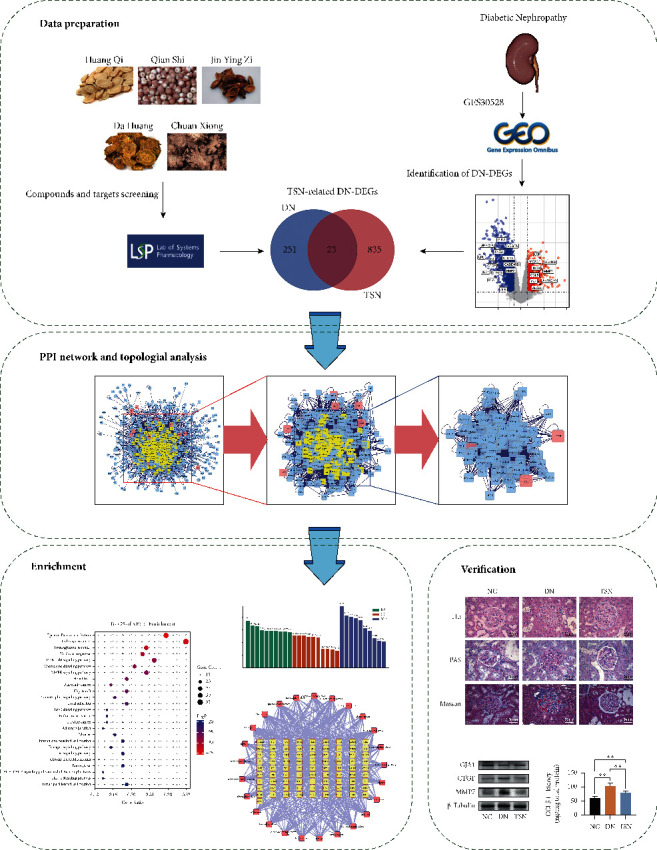
The schematic diagram of network pharmacological study of TSN for DN.

Currently, significant developments are taking place in the field of systems biology, in which network pharmacology is considered an important tool for drug discovery. Network pharmacology aims to describe a biological system using a network structure, switching the research paradigm to “network-targeted, multicomponent therapy.” This research model has similarities with TCM due to the synergistic mechanism of multicomponent therapeutic approaches that affect multiple pathways and therefore underlying mechanisms of TCM can be elucidated through network pharmacology [10, 11]. Therefore, we performed a systems biology-based approach to explore the underlying mechanisms of TSN in DN (Figure 1) as a guidance for further research.

## 2. Materials and Methods

### 2.1. Screening of TSN Compounds with Potential Biological Activity

TCMSP database (http://tcmspw.com/tcmsp.php) [12] was searched to obtain the candidate ingredients of the fives herbs contained in TSN. Then, compounds with oral bioavailability (OB) ≥30% and drug-likeness (DL) ≥0.18 were considered as potential bioactive ingredients [11, 13].

### 2.2. Target Screening

The TCMSP, PubChem database (https://pubchem.ncbi.nlm.nih.gov/) [14], and STITCH platform (http://stitch.embl.de/cgi/network.pl) [15] were used to search the targets of potential bioactive ingredients in TSN. In the PubChem database, targets were collected from the following four sections: Chemical-Gene Co-Occurrences in Literature, Protein Bound 3D Structures, Drug-Gene Interactions, and BioAssay Results. The threshold score was set to 0.9 to filter targets predicted by STITCH.

### 2.3. Identification of Differentially Expressed Genes (DEGs)

Gene expression data from 9 DN patients and 13 normal human glomeruli in GSE30528 Genest were downloaded from GEO database (https://www.ncbi.nlm.nih.gov/geo/), which were firstly contributed by Woroniecka et al. [16]. DEGs of glomeruli from DN patients and normal samples were obtained using the GEO2R online tool by setting |logFC| > 1.5 and *p* < 0.05. The gene expression was considered downregulated when the assay results indicated logFC < 0; if LogFC > 0, the gene expression was upregulated.

### 2.4. Network Construction

Networks were constructed using Cytoscape v3.8.0 [17] as follows: (1) component-target network, (2) protein-protein interaction (PPI) network for DEGs, (3) component-DEGs network, and (4) target-signaling pathway network.

### 2.5. PPI Network and Topological Analysis

TSN-related DEGs in DN (DN-DEGs) were identified as genes up- or downregulated in both TSN treatment and DN. The PPI network of TSN-related DN-DEGs was constructed using Bisogenet in Cytoscape. Three topology parameters, namely, degree, betweenness centrality, and closeness centrality, were chosen to analyze the topology of the network diagram, which reflected the topological importance of the nodes. Nodes with corresponding parameters greater than 2 times the median were selected to finally obtain the core targets of the PPI network [18]. “Degree” indicates how many edges were connected to a node; “betweenness centrality” is the times a node appears on the shortest path versus the total number of paths; “closeness centrality” measures the closeness of a node by calculating the distance of the shortest path from that node to the others [19].

### 2.6. Gene Ontology (GO) and KEGG Pathway Enrichment Analysis

The hub targets in the PPI network were analyzed using Metascape (http://metascape.org/gp/index.html) [20]. The enrichment results with *p* < 0.05 were ranked, and those with *p* < 0.01 were considered significantly enriched. Finally, the biological processes (BP), molecular functions (MF), cellular components (CC), and signaling pathways were identified.

### 2.7. Correlation of TSN-Related DN-DEGs with GFR

Nephroseq v5 (http://v5.nephroseq.org) is an online platform to mine comprehensive nephropathy gene expression datasets to identify markers of disease progression by correlating renal genetic phenotypes with known disease phenotypes [21]. Nephroseq v5 platform was applied to analyze the correlation between TSN-related DN-DEGs and GFR. TSN-related DN-DEGs were searched and the dataset Woroniecka Diabetes Glom, which is linked to GSE30528, was selected.

### 2.8. Experimental Validation

#### 2.8.1. Reagents

Streptozocin (STZ) was purchased from Sigma. (RANTES/CCL5) ELISA kit (CSB-E07398r) was purchased from Cusabio Biotech. Mouse monoclonal anti-CTGF antibody (Cat#: SC-365970) was purchased from Santa Cruz Biotechnology. Rabbit polyclonal anti-MMP7 antibodies (Cat#: 3801) and Rabbit polyclonal anti-GJA1 antibodies (Cat#: 3512) were purchased from Cell Signaling Technology. Mouse monoclonal anti-*β*-Tubulin antibody (Cat#: C1340) was purchased from APPLYGEN. Donkey Anti-Mouse IgG was purchased from Proteintech. Goat Anti-Rabbit IgG was purchased from LabLead.

#### 2.8.2. Animals and Models

Eighteen male Sprague Dawley rats (180–220 g) were purchased from Weitonglihua (Beijing, China). Rats were fed and given water ad libitum in an SPF environment. All procedures were approved by the Animal Experiments and Experimental Animal Welfare Committee of Capital Medical University.

Rats were randomly distributed, after one week of acclimatization, into normal control (NC, *n* = 6), DN group (DN, *n* = 6), and TSN group (TSN, *n* = 6). The DN rat model used in both DN and TSN groups was established as described previously [22]. Briefly, rats received intraperitoneal injections of streptozotocin (STZ, 55 mg/kg) and a high-fat diet (10% lard, 20% sucrose, 2.5% cholesterol, 0.5% sodium cholate, and 67% basic feed). Rats in NC were injected with the same dose of sodium citrate buffer and fed with a normal diet (12% fat, 28% protein, and 60% carbohydrate). After 7 days, random blood glucose (RGB) was measured in all rats in the diabetic group, and those rats with RBG levels above 16.7 mmol/L for three consecutive days were considered diabetic. Diabetic rats were randomly distributed into DN (DN, *n* = 6) and TSN (TSN, *n* = 6) groups. Rats in TSN group were administered 20g/kg TSN orally every day while rats in NC and DN were given the same volume of normal saline instead. The rats were euthanized after 12 weeks of treatment. Fresh kidneys were dissected and preserved under −80°C for further experiments.

#### 2.8.3. Histological Analysis

Formalin-fixed kidney tissues were embedded in paraffin and stained with hematoxylin-eosin (HE), Periodic Acid-Schiff (PAS), and Masson. The slices were analyzed and captured using a light microscope (Leica, DM4B) under ×400 magnification.

#### 2.8.4. ELISA Assay

CCL5 levels in the rat kidneys were measured using ELISA kit (Cusabio, Wuhan, China) according to the instructions provided.

#### 2.8.5. Western Blot Analysis

Kidney tissues were lysed and the supernatant was collected after centrifugation. After homogenization, protein symbol was separated using 10% SDS-PAGE electrophoresis and electrotransferred to PVDF membranes. Membranes were blocked with 5% non-fat milk and then incubated overnight at 4°C with the proper primary antibody for anti-GJA1 (Cell Signaling Technology, USA), CTGF (Santa Cruz Biotechnology, USA), MMP7 (Cell Signal Technology, USA), and *β*-Tubulin (APPLYGEN, China). After that, the membranes were incubated with corresponding secondary antibodies, including Donkey Anti-Mouse IgG (Proteintech, USA) and Goat Anti-Rabbit IgG (LabLead, China) at room temperature. Antigen-antibody immunoreactivity was visualized using electrochemiluminescence (ECL) reagents (Millipore, USA). Protein expressions were normalized according to the intensity of *β*-Tubulin and analyzed with Gelpro Analyzer software.

### 2.9. Statistical Analyses

Pearson's correlations between GFR and TSN-related DN-DEGs were performed using Nephroseq v5. Experimental data were presented as mean ± SD and analyzed using Graphpad prism 9.00. One-way ANOVA was applied for comparison between more than two groups. *p* < 0.05 means the comparison was statistically significant.

## 3. Results

### 3.1. Potential Bioactive Components and Targets in TSN

**Figure 2:**
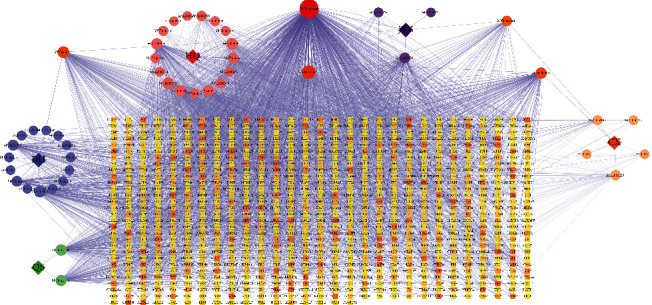
The Compound-Target network of TSN that consists of 910 nodes and 2912 edges. Circle and round square nodes denote the compounds and targets, respectively.

After screening with OB and DL value, 52 potential bioactive compounds were collected, including 20 from *Huang Qi*, 2 from *Qian Shi*, 16 from *Da Huang*, 7 from *Jin Yin Zi*, and 7 from *Chuan Xiong* and finally, 47 bioactive compounds were obtained by removing duplicates (Table 1). By searching these 47 components of TSN in TCMSP, PubChem, and STITCH databases, we obtained 858 corresponding potential targets. The Compound-Target network of TSN suggested potential synergistic effects of these herbs at shared targets (Figure 2).

### 3.2. DEGs Identified in DN

**Figure 3:**
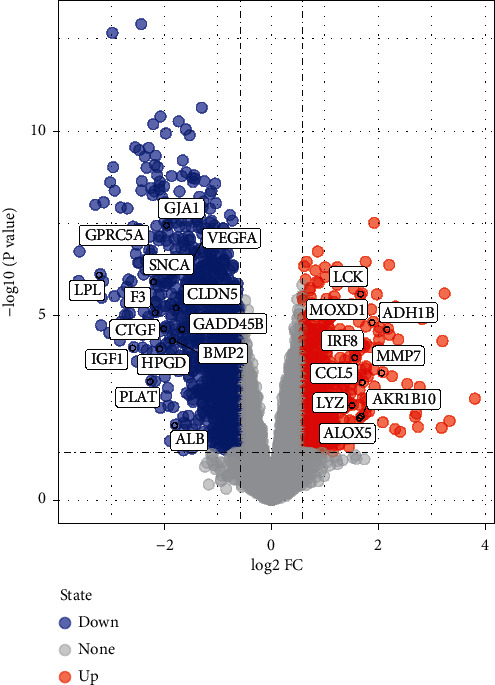
Volcano plot represents DEGs associated with diabetic nephropathy from GSE30528 dataset and 23 hub proteins targeted by TSN; blue plots represent downregulated DEGs; red plots represent upregulated genes.

Glomerular gene expression data from GSE30528 were analyzed, resulting in 274 DEGs (Figure 3 and Supplementary Table S1), including 67 upregulated (represented by red plots) and 207 downregulated genes (blue plots).

### 3.3. DN-DEGs Related to TSN

**Figure 4:**
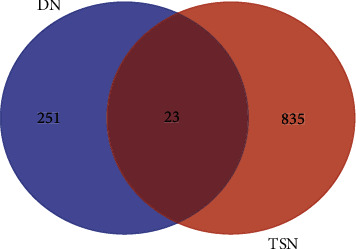
Venn diagram showing 23 TSN-related DN-DEGs.

**Figure 5:**
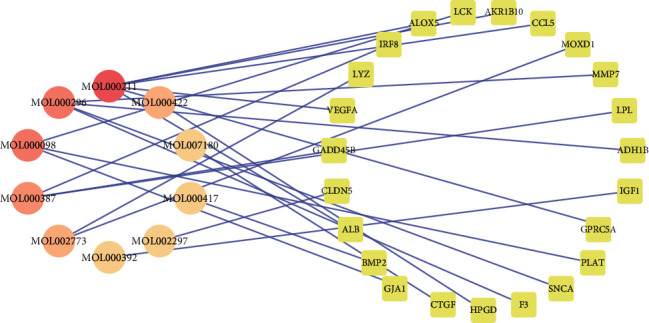
The network of compounds and 23 TSN-related DEGs. The circle and round square nodes indicate targets and compounds, respectively.

After mapping the DN-DEGs to 858 potential TSN targets, 23 common genes were collected as critical effect targets of TSN in DN (Figure 4) and this information is shown in Table 2. The expression levels of common targets provided by the matrix file of GSE30528 are shown in Supplementary Fig.  S1. A disease network including TCM compounds was constructed based on common targets (Figure 5), in which HQ contained the most active components associated with DN-DEGs, with a total of 7 targets, suggesting that HQ may be the most effective component in TSN. In addition, mairin (MOL000211) was related to the highest number of targets (7 in total), followed by quercetin (MOL000098) and hederagenin (MOL000296) (both with 5).

### 3.4. PPI Network Targets and Analysis

**Figure 6:**
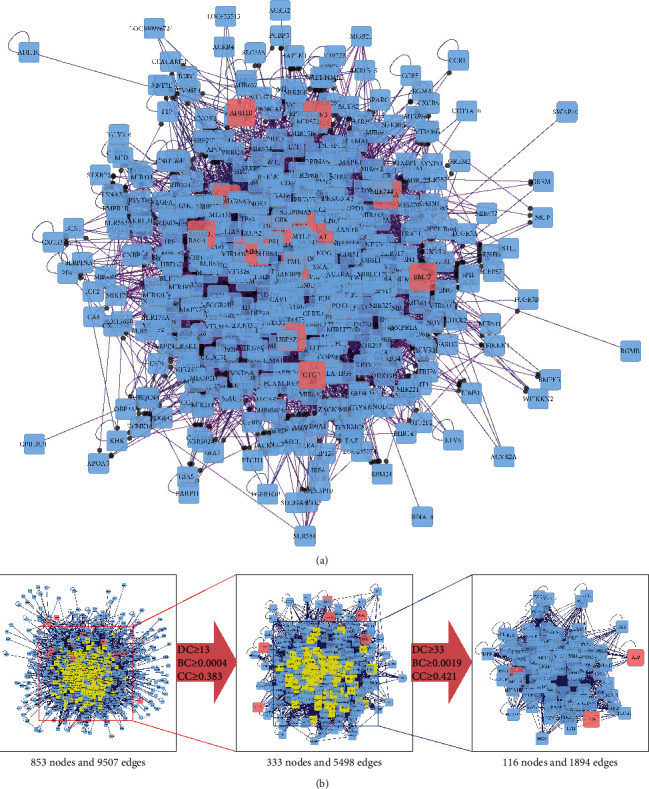
The construction and topological analysis of the PPI network. (a) The PPI network of TSN-related DN-DEGs was constructed using Bisogenet and the red nodes represent TSN-related DN-DEGs. (b) The process of topological analysis for the PPI network.

TSN-related DN-DEGs were imported into Bisogenet in Cytoscape to generate the PPI network. The network consisted of 883 nodes 9507 edges. The targets with “degree,” “betweenness centrality,” and “closeness centrality” above median values were selected as key targets. Details of the two screening and threshold setting are shown in Figure 6 and Supplementary Table S2. The screening results showed key underlying pathways of TSN in DN. A core PPI network consisting of 116 nodes and 1894 edges was constructed.

### 3.5. GO and KEGG Pathway Enrichment Analysis

**Figure 7:**
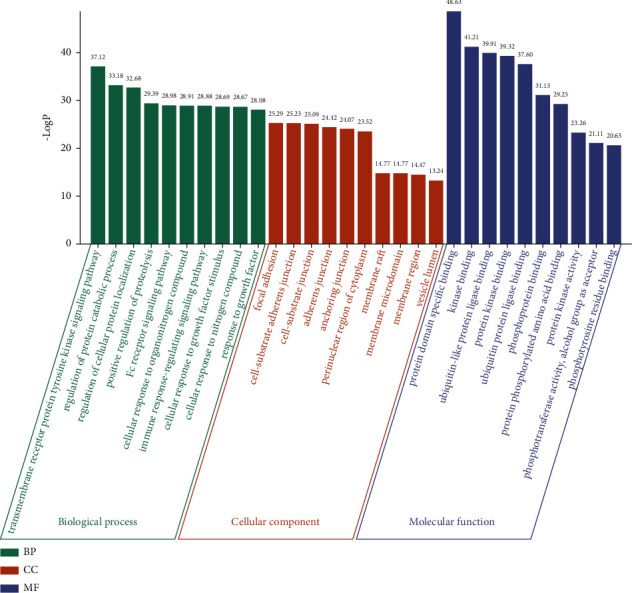
The GO enrichment of 116 key genes in PPI network. The top 10 items for each section were listed separately.

Enrichment analysis of the core network was performed using the Metascape platform to elucidate BP, CC, MF, and signaling pathways involved. GO enrichment analysis showed that the BP involved in TSN mainly included the transmembrane receptor protein tyrosine kinase signaling pathway, regulation of protein catabolic process, Fc receptor signaling pathway, immune response-regulating signaling pathway, cellular response to growth factor stimulus, and regulation of apoptotic signaling pathway. In addition, the above BPs were associated with related MFs, including protein domain specific binding, kinase binding, ubiquitin-like protein ligase binding, protein kinase binding, phosphoprotein binding, and protein phosphorylated amino acid binding. The major CCs involved focal adhesion, cell-substrate adherent junction, cell-substrate junction, perinuclear region of the cytoplasm, membrane region, and vesicle lumen (Figure 7).

**Figure 8:**
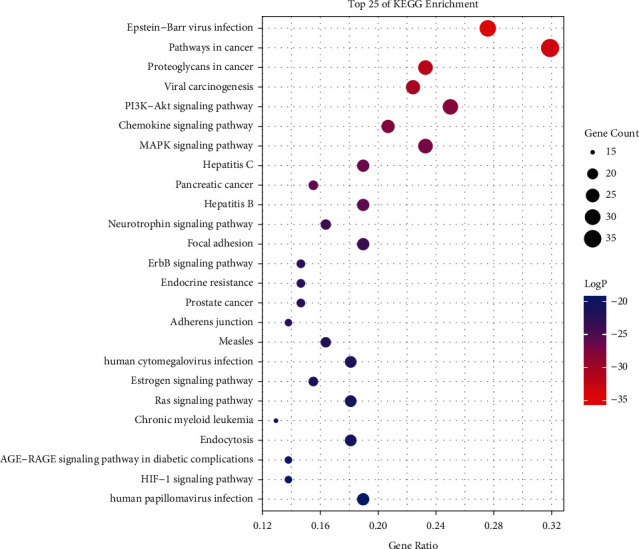
The KEGG enrichment analysis of the top 25 metabolic pathways.

**Figure 9:**
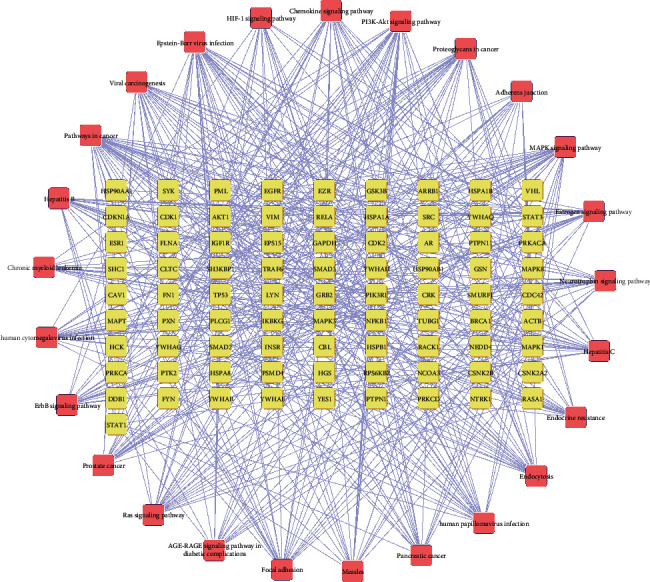
The network of targets involved in the major KEGG pathways. Circle and round square nodes denote the targets and signaling pathways, respectively.

165 signaling pathways were enriched according to enrichment analysis and the first 25 are shown in Figure 8. The main related pathways included PI3K-Akt, chemokine, MAPK, focal adhesion, ErbB, estrogen, Ras, AGE-RAGE, and HIF-1, endocrine resistance, and adherent junction signaling pathway. A network was constructed using Cytoscape for visualization to uncover the relationship between targets and pathways (Figure 9).

### 3.6. Association between DN-DEGs Related to TSN and Clinical Features of DN

The correlation between TSN-related DN-DEGs and GFR, the main clinical manifestation in DN, was investigated on the Nephroseq v5 (Supplementary Figure S2 and Table 3). There were no data for LZY and detailed information of other DEGs is shown in Supplementary Table S2. ALB, GPRC5A, PLAT, SNCA, F3, HPGD, CTGF, GJA1, BMP2, CLDN5, MODX1, GADD45B, and VEGFA were positively correlated with GFR, suggesting that these genes contributed to renal protection. In this case, the correlation between ALB and GFR was relatively weak (*R* = 0.454). Furthermore, IRF8, ALOX5, LCK, AKR1B10, CCL5, MOXD1, MMP7, and ADH1B were negatively correlated with GFR, implying that these genes were involved in the progression of DN. However, there was a nonlinear correlation between IGF1 and GFR (*p*=0.057).

### 3.7. Experimental Validation

#### 3.7.1. Kidney Histological Observations

**Figure 10:**
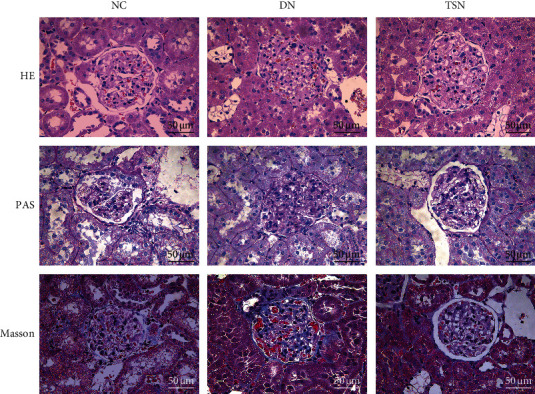
Renal histopathological changes in each group (magnification ×400).

HE, PAS, and Masson's staining results revealed that glomeruli from the NC group were of regular morphology, clear, and with a complete structure. Also, the mesangial membrane was not significantly thickened. There were hyperplasia and structural disorganization of glomerular cells in the DN group, with enlarged glomeruli and broadened mesangial matrix, which was accompanied by extensive collagen fibril formation and glycogen deposition. The histopathological damage in the kidney was improved in the TSN-treated rats, with relatively intact glomeruli structure and reduced fibrous deposition compared to those of DN animals (Figure 10).

#### 3.7.2. TSN Regulated the Expression of GJA1, CTGF, MMP7, and CCL5

**Figure 11:**
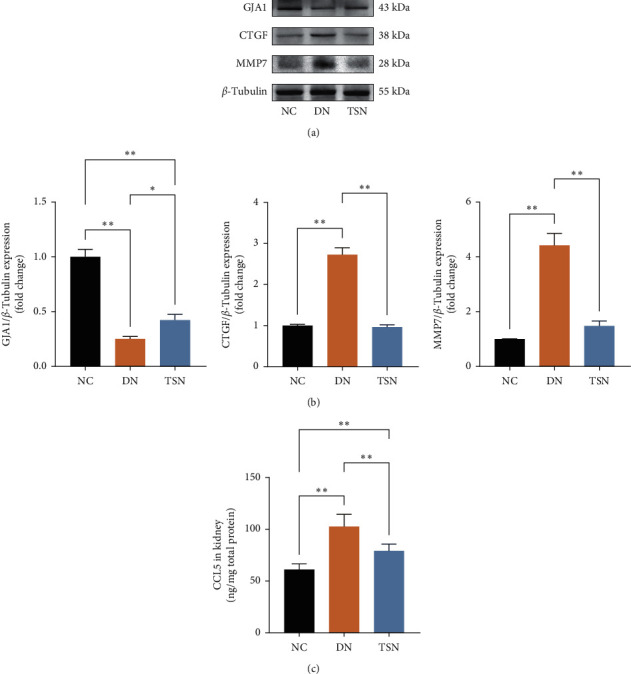
Effect of TSN on related DEGs in DN rats. Data are presented as the mean ± SD (^*∗*^*P* < 0.05; ^*∗*^*P* < 0.01). (a) Representative immunoblots for the CX43, CTGF, MMP7, and *β*-Tubulin proteins. (b) The relative expression levels of CX43/*β*-Tubulin and CTGF/*β*-Tubulin and MMP7/*β*-Tubulin. The data on quantified protein expressions were normalized by related *β*-Tubulin (fold change of NC). (c) CCL5s expression in all group.

In order to confirm the effect of TSN treatment in TSN-related DN-DEGs, the protein levels of GJA1, CTGF, and MMP7 were evaluated by western blot, and ELISA measured renal CCL5 levels. The results showed that the levels of CTGF, MMP7, and CCL5 level were increased in the DN group, while the GJA1 were decreased compared to the NC group. Indeed, TSN treatment attenuated the increase in CCL5, MMP7, and CTGF while it upregulated GJA1 (Figure 11).

## 4. Discussion

DN is an important microvascular complication of DM and also the main cause of ESRD. Indeed, DN is closely associated with increased mortality in DM patients. DN is multifactorial and is characterized by decreased GFR, proteinuria, and renal ultrastructural changes. Typical pathological changes in DN include tubular and glomerular basement membrane thickening, interstitial ECM expansion, glomerulosclerosis, and tubulointerstitial fibrosis, which ultimately cause renal hypofunction [23]. The main therapy for DN is to regulate blood glucose, blood lipids, and blood pressure [24]. However, the progressive decline of renal function in DN cannot be effectively prevented by glucose-lowing strategies. Therefore, the description of novel treatment strategies for DN is an important topic. In this regard, TCM has been used for many years in DM treatment and its complications [7, 24], being characterized as a multicomponent and multitarget strategy that acts on multiple pathways. TSN is an empirical formula for DN by a Chinese medicine expert, Yanbin Gao, which was effective in both humans and mice. In this study, the molecular network mechanism of TSN was explored based on network pharmacology to investigate potential underlying mechanisms of this formula used in DN.

In this study, we predicted the key herbs, active compounds, and potential effector targets in TSN. TSN-related DN-DEGs were obtained and found to be strongly correlated with decreases in GFR. GFR is an indicator of the extent of glomerular disease and the level of renal function. Therefore, these genes were identified as potential key targets of TSN treatment in DN. Besides, *HuangQi* and bioactive components mairin (degree = 7), quercetin (degree = 5), and hederagenin (degree = 5) were the key components in the treatment as they were high associated with these 23 targets. Mairin, also known as betulinic acid, can reduce glucose uptake and decrease endogenous glucose production [25, 26] by inhibiting alpha-glucosidase activity by competitively binding to alpha-glucosidase [27]. Mairin can also inhibit NF-*κ*B activation by preventing the degradation of I*κ*B in DN rats, resulting in the reduction of fibrosis in DN [28]. Quercetin not only provides improved renal function, but also possesses effects on antihyperglycemia and insulin resistance. Furthermore, quercetin reactivates the Hippo pathway to inhibit the proliferation of glomerular mesangial cells (MCs) in DN rats and MCs treated with high glucose, while quercetin also improves renal fibrosis and renal function in DN [29]. In addition, quercetin was shown to improve renal function in DN rats by downregulating TGF-*β*1 and CTGF [30, 31]. However, research on hederagenin is relatively scarce. As a dietary fiber, it has the potential effect to reduce lipid synthesis and lipid absorption in the intestine and promote the excretion of bile acids and triglycerides [32]. Although hyperlipidemia is an important factor in DM and DN, the effects of hederagenin in DM are still unclear. Network exploration reveals TSN's multicomponent synergistic effect on DN prevention.

In order to investigate the intrinsic mechanisms of TSN, we constructed a regulatory network of TSN-related DN-DEGs and performed the GO enrichment analysis. It was suggested that TSN may influence DM progression through multiple biological processes, including growth factor regulation, immune response, cellular stress response, and apoptosis. TSN may have a regulatory effect on focal adhesion, cell-substrate adherent junction, and cell-substrate junction, and these components are mainly participating in extracellular matrix (ECM) generation.

The pathways enriched are related to ECM production, cell proliferation and migration, inflammation, and endocrine disruption and suggested that the therapeutic efficacy of TSN is mediated by the synergy of multiple pathways. For example, AGEs can upregulate RAGE expression, activate multiple signaling pathways, including JAK-STAT, MAPK/ERK, PI3K/Akt/mTOR, and NF-*κ*B, and increase oxidative stress, inflammation, and renal fibrosis, causing structural and functional disorders in the kidney of patients with DM [33]. Active PI3K/AKT pathway in diabetic patients upregulates CTGF [34], which is involved in ECM deposition and promotes EMT in DN [35, 36]. Hyperglycemia also causes MAPK phosphorylation and activates the MAPK signaling pathway, the activation of which causes increased apoptosis, inflammatory cell invasion, and ECM synthesis [37]. Focal adhesion is involved in cell migration and ECM synthesis during EMT in DN [38]. Long-term overactivation of HIF-1*α* can induce the deposition in the ECM, causing glomerulosclerosis and renal interstitial fibrosis [39, 40]. Previous studies have reported that TSN exhibits a nephroprotective effect in DN mice by reversing podocyte EMT [9]. Combined with the results of GO and KEGG enrichment analysis, we presumed that a potential mechanism for TSN treatment in DN may be to reduce ECM deposition in the kidneys of DN patients by modulating levels of growth factors and chemokines, improving cellular stress and inflammation, and inhibiting EMT-induced cell proliferation.

EMT is an induced transformation of damaged cells into mesenchymal cells, with metastatic and fiber-generation capacity. The EMT process generates cytokines and chemokines, accompanied by the recruitment and proliferation of fibroblasts, causing the production and deposition of ECM, which participates in the progression of glomerulosclerosis and renal fibrosis [41–43]. Pathologically, DN presents itself with ECM accumulation and renal fibrosis. Extensive protein deposition in the basement membrane leads to thickening of the glomerular basement membrane, inducing interstitial fibrosis, glomerulosclerosis, and ultimately renal failure [41, 44]. Therefore, therapies directed at EMT and renal fibrosis are expected to be effective strategies for the treatment of DN. Through the establishment of the DN rat model, histopathological observation revealed that TSN effectively attenuated the extent of glomerular damage and reduced fibrous deposition, demonstrating a protective effect against renal fibrosis in DN rats.

The intrinsic mechanisms and related targets involved were further explored. Among the TSN-related DN-DEGs, GJA1, CTGF, MMP7, and CCL5 were our main targets of interest. Gap Junction Protein Alpha 1 (GJA1), also called CX43, is an essential component of cellular junctional structures during the transport of small molecules intercellularly. GJA1 prevents the proliferation of renal fibrosis in DN by improving oxidative stress [45] as well as downregulating TGF-*β*1 levels [46, 47]. The growth factor CTGF amplifies the fibrillogenic activity of TGF-*β*1 and induces the accumulation of extracellular matrix [48, 49]. MMP7, a member of the MMP family, can cleave a series of matrix and protein fragments, and release growth factors from the extracellular matrix, while promoting renal fibrosis [23, 50, 51]. In addition, the chemokine signaling pathway was also enriched in KEGG enrichment analysis. CCL5, a member of the C-C chemokine family, exerts a powerful chemotactic effect on immune cells, which is involved in chronic inflammatory seen in progression of DN [4, 52]. Therefore, the levels of CCL5 in each group were measured. As expected, GJA1 was upregulated and CTGF, MMP7, and CCL5 were downregulated by TSN to alleviate kidney fibrosis in DN, and these targets were demonstrated to be engaged in the treatment.

We investigated the role of TSN for DN therapy at the molecular level using a network pharmacology method and explored the possible mechanisms of its effect. The inhibition of renal fibrosis and inflammation by TSN is predicted to be a potential mechanism for the treatment of DN, and growth factors and chemokines may be key underlying mechanisms through which TSN exerts its nephroprotective effects.

## 5. Conclusion

In conclusion, potential active compounds, genes, and signaling pathways involved in the nephroprotection of TSN treatment were identified. TSN is perceived to be a promising strategy to treat DN through the synergistic effect of its “multicomponent, multitarget, and multipathway” compounds. Furthermore, it was verified that TSN could regulate the levels of GJA1, CTGF, MMP7, and CCL5 and alleviate renal fibrosis in DN. This work provides a science-based foundation for DN treatment with TSN. Additional experimental validation is essential to investigate other mechanisms involved in renal fibrosis and the role of the other TSN-related DEGs.

## Figures and Tables

**Table 1:** The potential bioactive compounds in TSN.

Mol ID	Compounds	OB	DL	Herb
MOL000211	Mairin	55.38	0.78	HQ
MOL000239	Jaranol	50.83	0.29	HQ
MOL000296	Hederagenin	36.91	0.75	HQ
MOL000033	(3S,8S,9S,10R,13R,14S,17R)-10,13-Dimethyl-17-[(2R,5S)-5-propan-2-yloctan-2-yl]-2,3,4,7,8,9,11,12,14,15,16,17-dodecahydro-1H-cyclopenta[a]phenanthren-3-ol	36.23	0.78	HQ
MOL000354	Isorhamnetin	49.6	0.31	HQ
MOL000371	3,9-Di-O-methylnissolin	53.74	0.48	HQ
MOL000374	5′-Hydroxyiso-muronulatol-2′,5′-di-O-glucoside	41.72	0.69	HQ
MOL000378	7-O-Methylisomucronulatol	74.69	0.3	HQ
MOL000379	9,10-Dimethoxypterocarpan-3-O-*β*-D-glucoside	36.74	0.92	HQ
MOL000380	(6aR,11aR)-9,10-Dimethoxy-6a,11a-dihydro-6H-benzofurano [3,2-c]chromen-3-ol	64.26	0.42	HQ
MOL000387	Bifendate	31.1	0.67	HQ
MOL000392	Formononetin	69.67	0.21	HQ
MOL000398	Isoflavanone	109.99	0.3	HQ
MOL000417	Calycosin	47.75	0.24	HQ
MOL000422	Kaempferol	41.88	0.24	HQ, JYZ
MOL000433	FA	68.96	0.71	HQ, CX
MOL000438	(3R)-3-(2-Hydroxy-3,4-dimethoxyphenyl)chroman-7-ol	67.67	0.26	HQ
MOL000439	Isomucronulatol-7,2′-di-O-glucosiole	49.28	0.62	HQ
MOL000442	1,7-Dihydroxy-3,9-dimethoxy pterocarpene	39.05	0.48	HQ
MOL000098	Quercetin	46.43	0.28	HQ, JYZ
MOL002773	Beta-carotene	37.18	0.58	QS
MOL007180	Vitamin-E	32.29	0.7	QS
MOL001494	Mandenol	42	0.19	JYZ, CX
MOL000358	Beta-sitosterol	36.91	0.75	JYZ, DH
MOL005030	Gondoic acid	30.7	0.2	JYZ
MOL008622	Methyl trametenolate	42.88	0.82	JYZ
MOL008628	4′-Methyl-N-methylcoclaurine	53.43	0.26	JYZ
MOL002280	Torachrysone-8-O-beta-D-(6′-oxayl)-glucoside	43.02	0.74	DH
MOL002281	Toralactone	46.46	0.24	DH
MOL002288	Emodin-1-O-beta-D-glucopyranoside	44.81	0.8	DH
MOL002293	Sennoside D_qt	61.06	0.61	DH
MOL002297	Daucosterol_qt	35.89	0.7	DH
MOL002303	Palmidin A	32.45	0.65	DH
MOL000471	Aloe-emodin	83.38	0.24	DH
MOL000554	Gallic acid-3-O-(6′-O-galloyl)-glucoside	30.25	0.67	DH
MOL000096	(−)-Catechin	49.68	0.24	DH
MOL002135	Myricanone	40.6	0.51	CX
MOL002140	Perlolyrine	65.95	0.27	CX
MOL002151	Senkyunone	47.66	0.24	CX
MOL002157	Wallichilide	42.31	0.71	CX
MOL000359	Sitosterol	36.91	0.75	CX
MOL002235	Eupatin	50.8	0.41	DH
MOL002251	Mutatochrome	48.64	0.61	DH
MOL002259	Physciondiglucoside	41.65	0.63	DH
MOL002260	Procyanidin B-5,3′-O-gallate	31.99	0.32	DH
MOL002268	Rhein	47.07	0.28	DH
MOL002276	Sennoside E_qt	50.69	0.61	DH

**Table 2:** The information of 23 TSN-related DN-DEGs.

Gene	Description	Log FC	*p* value	Regulation
LPL	Lipoprotein lipase	−3.19928	7.54*E* − 07	Downregulated
IGF1	Insulin-like growth factor 1	−2.57456	7.11*E* − 05	Downregulated
GPRC5A	G protein-coupled receptor class C group 5 member A	−2.27982	3.09*E* − 07	Downregulated
PLAT	Tissue-type plasminogen activator	−2.24543	0.000584	Downregulated
SNCA	Synuclein alpha	−2.1809	1.15*E* − 06	Downregulated
F3	Tissue factor	−2.14994	8.01*E* − 06	Downregulated
HPGD	15-Hydroxyprostaglandin dehydrogenase (NAD(+))	−2.06642	0.000075	Downregulated
CTGF	CCN family member 2	−1.98966	2.17*E* − 05	Downregulated
GJA1	Gap junction alpha-1 protein	−1.93855	3.39*E* − 08	Downregulated
BMP2	Bone morphogenetic protein 2	−1.82795	4.52*E* − 05	Downregulated
ALB	Albumin	−1.78623	0.00896	Downregulated
CLDN5	Claudin 5	−1.75802	5.85*E* − 06	Downregulated
GADD45B	Growth arrest and DNA damage inducible beta	−1.65127	2.25*E* − 05	Downregulated
VEGFA	Vascular endothelial growth factor A	−1.58624	5.92*E* − 07	Downregulated
LYZ	Lysozyme	1.529532	0.00258	Upregulated
IRF8	Interferon regulatory factor 8	1.583247	0.000131	Upregulated
ALOX5	Arachidonate 5-lipoxygenase	1.673735	0.00516	Upregulated
LCK	Tyrosine-protein kinase lck	1.694745	2.47*E* − 06	Upregulated
AKR1B10	Aldo-keto reductase family 1 member B10	1.705447	0.00508	Upregulated
CCL5	C-C motif chemokine receptor 5	1.720493	0.000615	Upregulated
MOXD1	Monooxygenase 1	1.905562	1.47*E* − 05	Upregulated
MMP7	Matrix metallopeptidase 7	2.08645	0.000339	Upregulated
ADH1B	Alcohol dehydrogenase 1B	2.183966	2.28*E* − 05	Upregulated

**Table 3:** Pearson's correlations between GFR and TSN-related DN-DEGs.

Target	*p* value	*R* value	*R* ^2^
MMP7	3.11*E* − 04	−0.697	0.485809
ADH1B	3.53*E* − 05	−0.764	0.583696
MOXD1	1.59*E* − 04	−0.72	0.5184
CCL5	0.001	−0.641	0.410881
AKR1B10	9.48*E* − 04	−0.655	0.429025
LCK	6.46*E* − 04	−0.67	0.4489
ALOX5	0.007	−0.557	0.310249
IRF8	0.014	−0.515	0.265225
VEGFA	2.00*E* − 05	0.778	0.605284
GADD45B	5.32*E* − 05	0.753	0.567009
CLDN5	2.06*E* − 05	0.778	0.605284
ALB	0.034	0.454	0.206116
BMP2	0.001	0.644	0.414736
GJA1	4.91*E* − 07	0.852	0.725904
CTGF	3.49*E* − 04	0.693	0.480249
HPGD	1.59*E* − 04	0.72	0.5184
F3	3.52*E* − 06	0.817	0.667489
SNCA	5.30*E* − 05	0.753	0.567009
PLAT	0.011	0.529	0.279841
GPRC5A	8.32*E* − 07	0.843	0.710649
IGF1	0.057	0.412	0.169744
LPL	0.0000137	0.787	0.619369

## Data Availability

The datasets used and/or analyzed during the current study are available from the corresponding author on reasonable request.

## Abbreviations

DN: Diabetic nephropathy
ESRD: End-stage renal disease
TSN: Tang-Shen-Ning decoction
GFR: Glomerular filtration rate
EMT: Epithelial-mesenchymal transition
ECM: Extracellular matrix
DEGs: Differentially expressed genes
PPI: Protein-protein interaction
RAAS: Renin-Angiotensin-Aldosterone system
TCM: Traditional Chinese Medicine
HQ: *Astragalus membranaceus* (Fisch.) Bunge
QS: *Euryale ferox* Salisb. ex DC
JYZ: *Rosa laevigata* Michx
DH: *Rheum officinale* Baill
CX: *Ligusticum chuanxiong* Hort
OB: Oral bioavailability
DL: Drug likeness
GO: Gene Ontology
BP: Biological processes
MF: Molecular functions
CC: Cellular components.

## Supplementary Materials

Supplementary Materials. Supplementary material 1: *Table S1*. The details of DN-DEGs in GSE30528. Supplementary material 2: *Table S2*. The information about topological parameters in the PPI network. Supplementary material 3: *Table S3*. The information of GFR and TSN-related DN-DEGs provided by Nephroseq v5. Supplementary material 4: *Figure S1*. Gene relative expression of TSN-related DN-DEGs in GSE30528. (A) 9 upregulated targets; (B) 14 downregulated targets. Supplementary material 5: *Figure S2*. Correlation between TSN-related DN-DEGs expression and GFR.
