# Supplementary figures and images for: Exploring the Potential Mechanism of Tang-Shen-Ning Decoction against Diabetic Nephropathy Based on the Combination of Network Pharmacology and Experimental Validation
Source: Evid Based Complement Alternat Med. 2021 Sep 9;2021:1025053. doi: 10.1155/2021/1025053 (PMC8445713; doi:10.1155/2021/1025053)

**A****up-regulated**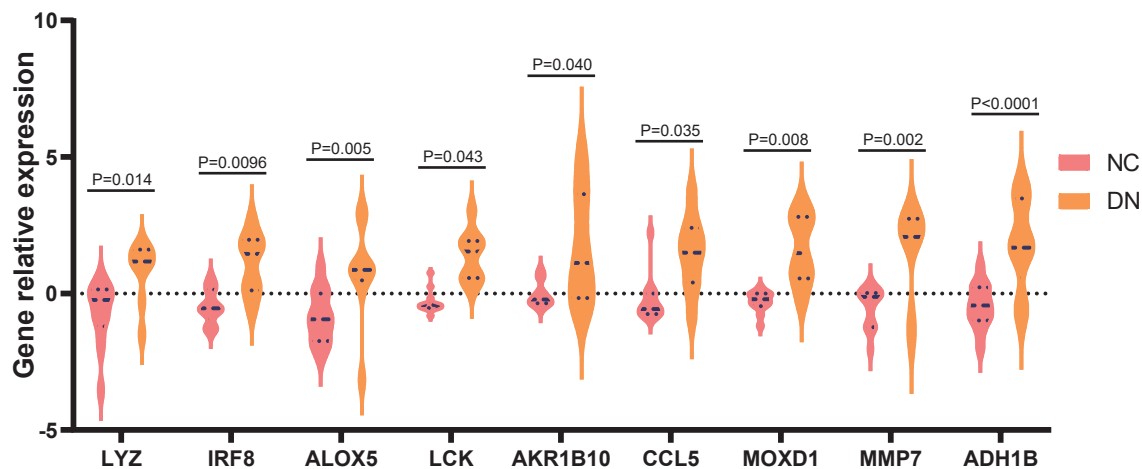**B****down-regulated**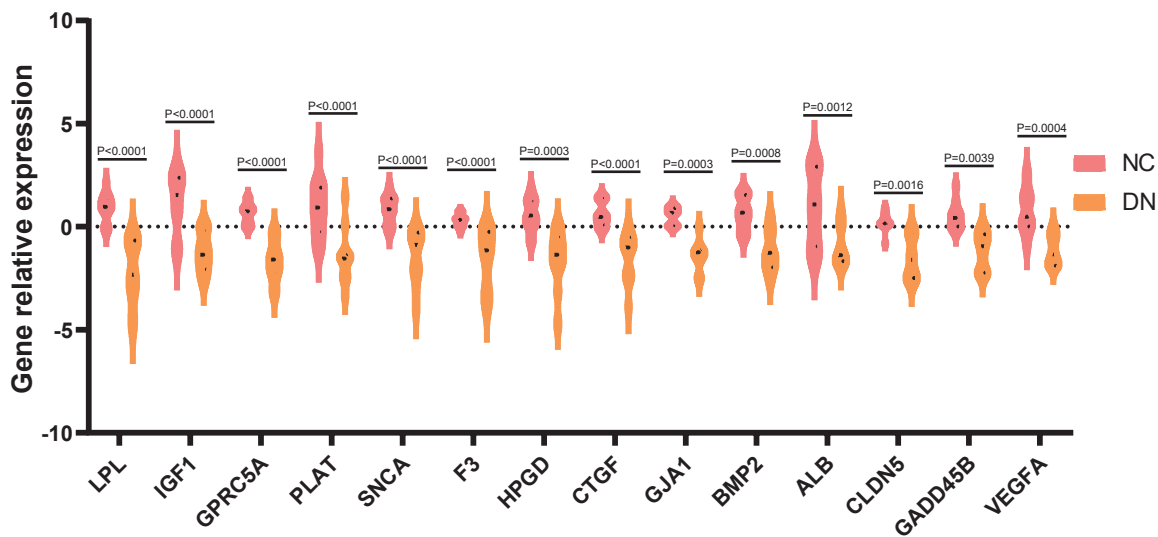

Supplement: Supplementary Materials. — Supplementary material 1: Table S1. The details of DN-DEGs in GSE30528. Supplementary material 2: Table S2. The information about topological parameters in the PPI network. Supplementary material 3: Table S3. The information of GFR and TSN-related DN-DEGs provided by Nephroseq v5. Supplementary material 4: Figure S1. Gene relative expression of TSN-related DN-DEGs in GSE30528. (A) 9 upregulated targets; (B) 14 downregulated targets. Supplementary material 5: Figure S2. Correlation between TSN-related DN-DEGs expression and GFR. [file 1025053.f1.zip › 1025053.f1/Fig S1.pdf]

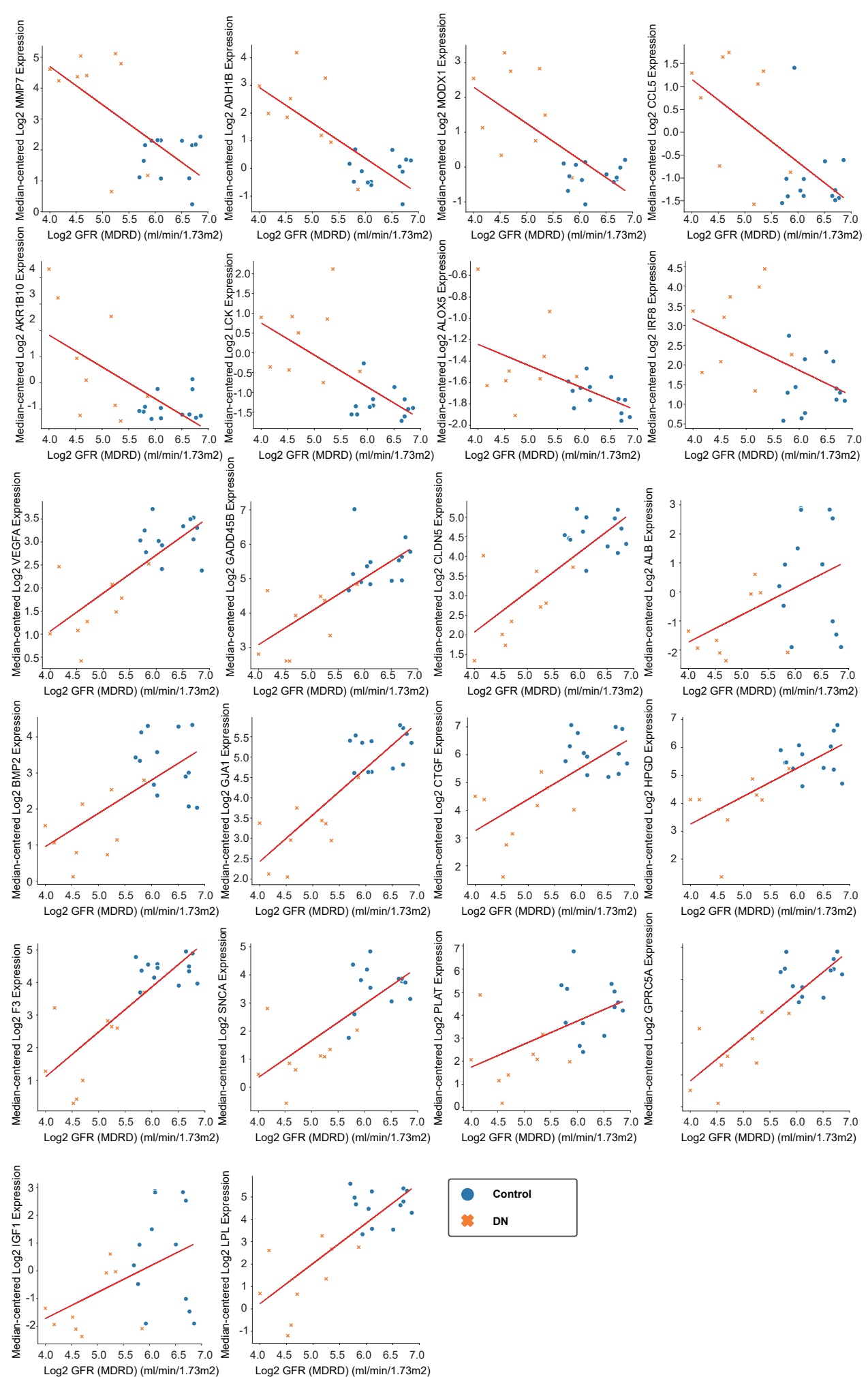

Supplement: Supplementary Materials. — Supplementary material 1: Table S1. The details of DN-DEGs in GSE30528. Supplementary material 2: Table S2. The information about topological parameters in the PPI network. Supplementary material 3: Table S3. The information of GFR and TSN-related DN-DEGs provided by Nephroseq v5. Supplementary material 4: Figure S1. Gene relative expression of TSN-related DN-DEGs in GSE30528. (A) 9 upregulated targets; (B) 14 downregulated targets. Supplementary material 5: Figure S2. Correlation between TSN-related DN-DEGs expression and GFR. [file 1025053.f1.zip › 1025053.f1/Fig S2.pdf]
