# Supplementary material for: Exploring the Potential Mechanism of Tang-Shen-Ning Decoction against Diabetic Nephropathy Based on the Combination of Network Pharmacology and Experimental Validation
Source: Evid Based Complement Alternat Med. 2021 Sep 9;2021:1025053. doi: 10.1155/2021/1025053 (PMC8445713; doi:10.1155/2021/1025053)
Supplement: Supplementary Materials. — Supplementary material 1: Table S1. The details of DN-DEGs in GSE30528. Supplementary material 2: Table S2. The information about topological parameters in the PPI network. Supplementary material 3: Table S3. The information of GFR and TSN-related DN-DEGs provided by Nephroseq v5. Supplementary material 4: Figure S1. Gene relative expression of TSN-related DN-DEGs in GSE30528. (A) 9 upregulated targets; (B) 14 downregulated targets. Supplementary material 5: Figure S2. Correlation between TSN-related DN-DEGs expression and GFR. [file 1025053.f1.zip › 1025053.f1/Table S1.doc]

| **The details of DN-DEGs in GSE30528** | | | | |
| --- | --- | --- | --- | --- |
| **Gene symbol** | **ID** | **Gene title** | **log FC** | **P Value** |
| ADH1B | 209613_s_at | alcohol dehydrogenase 1B (class I), beta polypeptide | 2.2957 | 1E-04 |
| AIF1 | 209901_x_at | allograft inflammatory factor 1 | -1.6724 | 8E-07 |
| GALC | 204417_at | galactosylceramidase | -1.7344 | 5E-11 |
| TRAM2 | 202369_s_at | translocation associated membrane protein 2 | -2.2102 | 6E-11 |
| ZNF415 | 205514_at | zinc finger protein 415 | -1.5987 | 9E-11 |
| PARVA | 213675_at | parvin alpha | -1.9745 | 1E-10 |
| ARHGEF10 | 216620_s_at | Rho guanine nucleotide exchange factor 10 | -1.5285 | 1E-10 |
| CA10 | 220889_s_at | carbonic anhydrase 10 | -2.5468 | 3E-10 |
| CERS6 | 212446_s_at | ceramide synthase 6 | -2.1656 | 5E-10 |
| DPYSL3 | 201431_s_at | dihydropyrimidinase like 3 | -2.3693 | 5E-10 |
| MPP5 | 219321_at | membrane palmitoylated protein 5 | -1.6583 | 6E-10 |
| TCF7L1 | 221016_s_at | transcription factor 7 like 1 | -2.1805 | 8E-10 |
| C1orf21 | 221272_s_at | chromosome 1 open reading frame 21 | -2.1294 | 9E-10 |
| NPHS1 | 207673_at | NPHS1, nephrin | -2.9627 | 1E-09 |
| ZNF185 | 203585_at | zinc finger protein 185 (LIM domain) | -2.3358 | 1E-09 |
| APBB2 | 212970_at | amyloid beta precursor protein binding family B member 2 | -1.7261 | 4E-08 |
| CCDC91 | 218545_at | coiled-coil domain containing 91 | -1.8674 | 2E-09 |
| ARHGAP19 | 37577_at | Rho GTPase activating protein 19 | -2.4702 | 3E-10 |
| ARL4C | 202206_at | ADP ribosylation factor like GTPase 4C | 1.5662 | 2E-04 |
| PRKAR2B | 203680_at | protein kinase cAMP-dependent type II regulatory subunit beta | -3.0161 | 2E-09 |
| CACNB2 | 213714_at | calcium voltage-gated channel auxiliary subunit beta 2 | -2.0981 | 3E-09 |
| FBXO9 | 212991_at | F-box protein 9 | -2.0071 | 3E-09 |
| TGFBR3 | 204731_at | transforming growth factor beta receptor 3 | -2.2504 | 4E-09 |
| KLF7 | 204334_at | Kruppel like factor 7 | -2.0937 | 4E-09 |
| ARRB1 | 49111_at | arrestin beta 1 | -1.6735 | 2E-09 |
| FAM134B | 218510_x_at | family with sequence similarity 134 member B | -2.1916 | 4E-09 |
| FBXL7 | 213249_at | F-box and leucine rich repeat protein 7 | -1.7178 | 4E-09 |
| CORO2B | 209789_at | coronin 2B | -2.045 | 6E-09 |
| B3GALT2 | 217452_s_at | beta-1,3-galactosyltransferase 2 | -2.4244 | 2E-09 |
| ARHGEF12 | 201335_s_at | Rho guanine nucleotide exchange factor 12 | -1.985 | 8E-09 |
| DPP6 | 207789_s_at | dipeptidyl peptidase like 6 | -2.8181 | 1E-08 |
| PDPN | 221898_at | podoplanin | -2.6944 | 1E-08 |
| PNMA2 | 209598_at | paraneoplastic Ma antigen 2 | -1.8495 | 2E-08 |
| BMP2 | 205289_at | bone morphogenetic protein 2 | -1.828 | 5E-05 |
| CXADR | 203917_at | coxsackie virus and adenovirus receptor | -1.6865 | 3E-08 |
| CTDSPL | 201906_s_at | CTD small phosphatase like | -1.5527 | 3E-08 |
| BMP7 | 209590_at | bone morphogenetic protein 7 | -2.0877 | 2E-09 |
| C1QA | 218232_at | complement C1q A chain | 1.9223 | 3E-08 |
| GJA1 | 201667_at | gap junction protein alpha 1 | -1.9386 | 3E-08 |
| LOC728392 | 218380_at | uncharacterized LOC728392 | -1.9527 | 4E-08 |
| KLK6 | 204733_at | kallikrein related peptidase 6 | -2.5896 | 4E-08 |
| BTG3 | 213134_x_at | BTG anti-proliferation factor 3 | -1.5932 | 1E-09 |
| CD163 | 215049_x_at | CD163 molecule | 1.9733 | 3E-06 |
| TNNT2 | 215389_s_at | troponin T2, cardiac type | -3.2378 | 6E-08 |
| CDC42EP3 | 209286_at | CDC42 effector protein 3 | -2.3525 | 3E-07 |
| ERP44 | 208958_at | endoplasmic reticulum protein 44 | -1.547 | 7E-08 |
| CD200 | 209583_s_at | CD200 molecule | -2.1052 | 7E-08 |
| NRIP2 | 215104_at | nuclear receptor interacting protein 2 | -1.742 | 7E-08 |
| LRRC2 | 219949_at | leucine rich repeat containing 2 | -3.1681 | 8E-08 |
| MME | 203435_s_at | membrane metallo-endopeptidase | -1.6565 | 8E-08 |
| CDKN1C | 219534_x_at | cyclin dependent kinase inhibitor 1C | -2.081 | 8E-06 |
| SEPTIN11 | 201307_at | septin 11 | -1.5163 | 8E-08 |
| KBTBD11 | 204301_at | kelch repeat and BTB domain containing 11 | -1.5451 | 9E-08 |
| H2AFJ | 220936_s_at | H2A histone family member J | -2.1094 | 9E-08 |
| CHI3L1 | 209396_s_at | chitinase 3 like 1 | -3.5874 | 2E-07 |
| MDFIC | 211675_s_at | MyoD family inhibitor domain containing | -1.6496 | 1E-07 |
| SYNPO | 202796_at | synaptopodin | -2.5101 | 1E-07 |
| F2R | 203989_x_at | coagulation factor II thrombin receptor | -1.9205 | 1E-07 |
| GULP1 | 204235_s_at | GULP, engulfment adaptor PTB domain containing 1 | -1.619 | 1E-07 |
| RECK | 205407_at | reversion inducing cysteine rich protein with kazal motifs | -1.6878 | 1E-07 |
| CLIC5 | 217628_at | chloride intracellular channel 5 | -1.9974 | 2E-06 |
| ZFPM2 | 219778_at | zinc finger protein, FOG family member 2 | -1.5084 | 2E-07 |
| SYNJ2 | 212828_at | synaptojanin 2 | -2.2365 | 2E-07 |
| ZDHHC6 | 218249_at | zinc finger DHHC-type containing 6 | -2.256 | 2E-07 |
| TCF21 | 204931_at | transcription factor 21 | -2.5398 | 2E-07 |
| ARHGEF3 | 218501_at | Rho guanine nucleotide exchange factor 3 | -1.7953 | 3E-07 |
| CTNNBIP1 | 203081_at | catenin beta interacting protein 1 | -1.5055 | 3E-07 |
| MCM6 | 201930_at | minichromosome maintenance complex component 6 | -1.6442 | 3E-07 |
| CLU | 208792_s_at | clusterin | 1.8286 | 0.004 |
| DLC1 | 210762_s_at | DLC1 Rho GTPase activating protein | -1.7747 | 3E-07 |
| MXRA8 | 213422_s_at | matrix remodeling associated 8 | -1.6808 | 3E-07 |
| CDC14A | 205288_at | cell division cycle 14A | -2.3459 | 3E-07 |
| SERPINE2 | 212190_at | serpin family E member 2 | 1.7635 | 3E-07 |
| SRGAP2C | 213329_at | SLIT-ROBO Rho GTPase activating protein 2C///SLIT-ROBO Rho GTPase activating protein 2B///SLIT-ROBO Rho GTPase activating protein 2 | -2.4758 | 4E-07 |
| STON1 | 213413_at | stonin 1 | -1.5354 | 4E-07 |
| COL1A2 | 202404_s_at | collagen type I alpha 2 chain | 2.591 | 9E-06 |
| AKT3 | 212609_s_at | AKT serine/threonine kinase 3 | -1.5421 | 4E-07 |
| C1QB | 202953_at | complement C1q B chain | 2.2047 | 4E-07 |
| ITGB5 | 201124_at | integrin subunit beta 5 | -2.0664 | 5E-07 |
| FGF9 | 206404_at | fibroblast growth factor 9 | -1.6282 | 5E-07 |
| MGAT5 | 212098_at | mannosyl (alpha-1,6-)-glycoprotein beta-1,6-N-acetyl-glucosaminyltransferase | -2.3528 | 6E-07 |
| GRK5 | 204396_s_at | G protein-coupled receptor kinase 5 | -1.8399 | 6E-07 |
| GAS1 | 204457_s_at | growth arrest specific 1 | -2.0487 | 6E-07 |
| TPM2 | 204083_s_at | tropomyosin 2 (beta) | -2.1239 | 6E-07 |
| ELOVL4 | 219532_at | ELOVL fatty acid elongase 4 | -1.8619 | 6E-07 |
| CR1 | 217484_at | complement component 3b/4b receptor 1 (Knops blood group) | -1.7457 | 8E-06 |
| PDE4B | 203708_at | phosphodiesterase 4B | -1.5112 | 7E-07 |
| NTNG1 | 206713_at | netrin G1 | -1.88 | 7E-07 |
| ETS2 | 201328_at | ETS proto-oncogene 2, transcription factor | -1.8875 | 8E-07 |
| DACH1 | 205471_s_at | dachshund family transcription factor 1 | -2.2228 | 5E-09 |
| EXPH5 | 213929_at | exophilin 5 | -2.153 | 4E-08 |
| HPS5 | 204544_at | HPS5, biogenesis of lysosomal organelles complex 2 subunit 2 | -1.8177 | 1E-06 |
| WT1-AS | 206954_at | WT1 antisense RNA | -1.8651 | 1E-06 |
| SPOCK2 | 202524_s_at | sparc/osteonectin, cwcv and kazal-like domains proteoglycan (testican) 2 | -2.1013 | 1E-06 |
| F5 | 204714_s_at | coagulation factor V | -2.4924 | 5E-08 |
| FAM65A | 45749_at | family with sequence similarity 65 member A | -1.7912 | 2E-07 |
| GRIK2 | 213845_at | glutamate ionotropic receptor kainate type subunit 2 | -1.8852 | 1E-06 |
| FGF1 | 208240_s_at | fibroblast growth factor 1 | -1.8697 | 1E-05 |
| FN1 | 210495_x_at | fibronectin 1 | 1.5403 | 2E-06 |
| MAFB | 218559_s_at | MAF bZIP transcription factor B | -1.9384 | 1E-06 |
| FRY | 204072_s_at | FRY microtubule binding protein | -2.6767 | 1E-06 |
| FYN | 212486_s_at | FYN proto-oncogene, Src family tyrosine kinase | -1.9984 | 3E-08 |
| KLK7 | 205778_at | kallikrein related peptidase 7 | -2.1131 | 2E-06 |
| SEMA3G | 219689_at | semaphorin 3G | -2.7059 | 2E-06 |
| MYO1B | 212364_at | myosin IB | -1.7933 | 2E-06 |
| GPRC5A | 203108_at | G protein-coupled receptor class C group 5 member A | -2.2798 | 3E-07 |
| FOXC1 | 213260_at | forkhead box C1 | -1.629 | 2E-06 |
| TPPP3 | 218876_at | tubulin polymerization promoting protein family member 3 | -2.7828 | 2E-06 |
| LCK | 204891_s_at | LCK proto-oncogene, Src family tyrosine kinase | 1.6947 | 2E-06 |
| RARRES1 | 221872_at | retinoic acid receptor responder 1 | 3.2444 | 3E-06 |
| HTRA1 | 201185_at | HtrA serine peptidase 1 | -1.8647 | 3E-06 |
| ZNF804A | 215767_at | zinc finger protein 804A | -2.2549 | 3E-06 |
| RASL11B | 219142_at | RAS like family 11 member B | -2.0589 | 3E-06 |
| RASGRP3 | 205801_s_at | RAS guanyl releasing protein 3 | -1.6744 | 3E-06 |
| COLGALT2 | 209883_at | collagen beta(1-O)galactosyltransferase 2 | -1.6848 | 3E-06 |
| JAM3 | 212813_at | junctional adhesion molecule 3 | -1.6092 | 3E-06 |
| NFASC | 213438_at | neurofascin | -2.9395 | 3E-06 |
| HPGD | 211548_s_at | hydroxyprostaglandin dehydrogenase 15-(NAD) | -2.0664 | 8E-05 |
| GHR | 205498_at | growth hormone receptor | -1.8007 | 3E-06 |
| IGF1 | 209541_at | insulin like growth factor 1 | -2.5746 | 7E-05 |
| SMAD6 | 207069_s_at | SMAD family member 6 | -1.706 | 4E-06 |
| MIR6872 | 203071_at | microRNA 6872///semaphorin 3B | -1.5884 | 4E-06 |
| ZNF91 | 206059_at | zinc finger protein 91 | -2.1692 | 4E-06 |
| NES | 218678_at | nestin | -2.5155 | 4E-06 |
| HS3ST3A1 | 219985_at | heparan sulfate-glucosamine 3-sulfotransferase 3A1 | -2.1961 | 4E-06 |
| CAND2 | 213547_at | cullin associated and neddylation dissociated 2 (putative) | -1.5464 | 5E-06 |
| SPOCK1 | 202363_at | sparc/osteonectin, cwcv and kazal-like domains proteoglycan (testican) 1 | -2.724 | 5E-06 |
| TSPAN2 | 214606_at | tetraspanin 2 | -1.8798 | 5E-06 |
| IGK | 221651_x_at | immunoglobulin kappa locus///immunoglobulin kappa constant | 2.7133 | 0.004 |
| VSIG4 | 204787_at | V-set and immunoglobulin domain containing 4 | 1.5767 | 5E-06 |
| AMIGO2 | 222108_at | adhesion molecule with Ig like domain 2 | -2.1694 | 5E-06 |
| TBXA2R | 336_at | thromboxane A2 receptor | -1.9933 | 5E-06 |
| ZNF124 | 206928_at | zinc finger protein 124 | -1.6719 | 6E-06 |
| SERPINA3 | 202376_at | serpin family A member 3 | 2.3221 | 6E-06 |
| CLDN5 | 204482_at | claudin 5 | -1.758 | 6E-06 |
| IGLC1 | 209138_x_at | immunoglobulin lambda constant 1 | 2.7439 | 0.01 |
| MYLIP | 220319_s_at | myosin regulatory light chain interacting protein | -1.5839 | 7E-06 |
| MS4A6A | 219666_at | membrane spanning 4-domains A6A | 1.869 | 7E-06 |
| MEGF9 | 212830_at | multiple EGF like domains 9 | -1.506 | 7E-06 |
| PTGER4 | 204897_at | prostaglandin E receptor 4 | -1.8059 | 7E-06 |
| PDLIM2 | 219165_at | PDZ and LIM domain 2 | -1.5035 | 7E-06 |
| LGALS8 | 208933_s_at | galectin 8 | -2.4355 | 1E-13 |
| ATP10A | 214255_at | ATPase phospholipid transporting 10A (putative) | -2.3912 | 8E-06 |
| TCEAL2 | 211276_at | transcription elongation factor A like 2 | -2.1919 | 8E-06 |
| F3 | 204363_at | coagulation factor III, tissue factor | -2.1499 | 8E-06 |
| TM2D1 | 213882_at | TM2 domain containing 1 | -1.5108 | 9E-06 |
| ARHGAP28 | 220382_s_at | Rho GTPase activating protein 28 | -1.869 | 9E-06 |
| LOC101928916 | 202238_s_at | uncharacterized LOC101928916///nicotinamide N-methyltransferase | 2.1222 | 6E-06 |
| LOC101930416 | 213388_at | myomegalin-like///uncharacterized LOC101929792///phosphodiesterase 4D interacting protein-like///phosphodiesterase 4D interacting protein | -2.0644 | 7E-08 |
| LOX | 204298_s_at | lysyl oxidase | -2.9772 | 2E-13 |
| TRIB2 | 202478_at | tribbles pseudokinase 2 | -1.5598 | 1E-05 |
| LPL | 203549_s_at | lipoprotein lipase | -3.1993 | 8E-07 |
| MOXD1 | 209708_at | monooxygenase DBH like 1 | 1.9056 | 1E-05 |
| WT1 | 206067_s_at | Wilms tumor 1 | -2.3169 | 2E-05 |
| MAGI2 | 209737_at | membrane associated guanylate kinase, WW and PDZ domain containing 2 | -3.1383 | 8E-09 |
| COL4A5 | 213110_s_at | collagen type IV alpha 5 chain | -1.8065 | 2E-05 |
| NDNF | 219747_at | neuron derived neurotrophic factor | -3.1851 | 2E-05 |
| EPB41L5 | 220977_x_at | erythrocyte membrane protein band 4.1 like 5 | -1.5168 | 2E-05 |
| MYLK3 | 217623_at | myosin light chain kinase 3 | -2.7198 | 2E-05 |
| MYL9 | 201058_s_at | myosin light chain 9 | -1.9794 | 2E-05 |
| VTCN1 | 219768_at | V-set domain containing T cell activation inhibitor 1 | 1.7686 | 2E-05 |
| CPA3 | 205624_at | carboxypeptidase A3 | 2.031 | 2E-05 |
| ITIH5 | 219064_at | inter-alpha-trypsin inhibitor heavy chain family member 5 | -1.9145 | 2E-05 |
| C14orf132 | 218820_at | chromosome 14 open reading frame 132 | -1.6033 | 2E-05 |
| CTGF | 209101_at | connective tissue growth factor | -1.9897 | 2E-05 |
| GADD45B | 207574_s_at | growth arrest and DNA damage inducible beta | -1.6513 | 2E-05 |
| KCND3 | 213832_at | potassium voltage-gated channel subfamily D member 3 | -1.7971 | 2E-05 |
| DCN | 211896_s_at | decorin | -1.6764 | 2E-05 |
| APOD | 201525_at | apolipoprotein D | -2.3336 | 2E-05 |
| EMCN | 219436_s_at | endomucin | -2.0876 | 3E-05 |
| C7 | 202992_at | complement component 7 | 2.0071 | 3E-05 |
| PCOLCE2 | 219295_s_at | procollagen C-endopeptidase enhancer 2 | -3.0358 | 3E-05 |
| ID4 | 209292_at | inhibitor of DNA binding 4, HLH protein | -1.7736 | 3E-05 |
| COL6A3 | 201438_at | collagen type VI alpha 3 chain | 2.2093 | 3E-05 |
| MARCKS | 201670_s_at | myristoylated alanine rich protein kinase C substrate | 1.5074 | 5E-05 |
| AZIN1 | 213747_at | antizyme inhibitor 1 | -1.8471 | 3E-05 |
| HRG | 206226_at | histidine rich glycoprotein | -1.6436 | 4E-05 |
| GOLIM4 | 204324_s_at | golgi integral membrane protein 4 | -1.8938 | 4E-05 |
| TYRO3 | 211432_s_at | TYRO3 protein tyrosine kinase | -1.5879 | 4E-05 |
| THBS2 | 203083_at | thrombospondin 2 | 2.014 | 4E-05 |
| LUM | 201744_s_at | lumican | 2.3746 | 4E-05 |
| MYOZ2 | 213782_s_at | myozenin 2 | -1.9589 | 1E-06 |
| PTPRO | 208121_s_at | protein tyrosine phosphatase, receptor type O | -2.8369 | 5E-05 |
| LTF | 202018_s_at | lactotransferrin | 3.2036 | 5E-05 |
| NEBL | 203962_s_at | nebulette | -3.1509 | 1E-06 |
| SLC14A1 | 205856_at | solute carrier family 14 member 1 (Kidd blood group) | -1.5201 | 5E-05 |
| LEF1 | 221558_s_at | lymphoid enhancer binding factor 1 | 1.6107 | 5E-05 |
| PLA2R1 | 207415_at | phospholipase A2 receptor 1 | -2.585 | 3E-06 |
| PSD3 | 203355_s_at | pleckstrin and Sec7 domain containing 3 | -1.5957 | 6E-05 |
| CD53 | 203416_at | CD53 molecule | 1.6721 | 6E-05 |
| PLCE1 | 205112_at | phospholipase C epsilon 1 | -3.2902 | 1E-08 |
| CFB | 202357_s_at | complement factor B | 1.7204 | 6E-05 |
| CD48 | 204118_at | CD48 molecule | 1.7367 | 7E-05 |
| EVI2A | 204774_at | ecotropic viral integration site 2A | 1.8399 | 7E-05 |
| PTGDS | 211748_x_at | prostaglandin D2 synthase | -2.649 | 9E-06 |
| IL13RA2 | 206172_at | interleukin 13 receptor subunit alpha 2 | -2.4093 | 7E-05 |
| EML1 | 204796_at | echinoderm microtubule associated protein like 1 | -1.5624 | 7E-05 |
| APOLD1 | 221031_s_at | apolipoprotein L domain containing 1 | -2.0601 | 8E-05 |
| FZD2 | 210220_at | frizzled class receptor 2 | -1.8398 | 8E-05 |
| EHD3 | 218935_at | EH domain containing 3 | -2.3345 | 9E-05 |
| CD3D | 213539_at | CD3d molecule | 1.6492 | 9E-05 |
| COL4A3 | 222073_at | collagen type IV alpha 3 chain | -1.869 | 9E-05 |
| CD52 | 34210_at | CD52 molecule | 2.2327 | 1E-04 |
| PALLD | 200906_s_at | palladin, cytoskeletal associated protein | -1.5448 | 1E-04 |
| SEMA5A | 205405_at | semaphorin 5A | -2.4304 | 4E-09 |
| DDN | 214788_x_at | dendrin | -1.9036 | 1E-04 |
| SNCA | 204467_s_at | synuclein alpha | -2.1809 | 1E-06 |
| TDO2 | 205943_at | tryptophan 2,3-dioxygenase | 1.5665 | 1E-04 |
| IRF8 | 204057_at | interferon regulatory factor 8 | 1.5832 | 1E-04 |
| HIST1H2BK | 209806_at | histone cluster 1, H2bk | -1.5944 | 1E-04 |
| C3 | 217767_at | complement component 3 | 2.68 | 2E-04 |
| PAMR1 | 213661_at | peptidase domain containing associated with muscle regeneration 1 | -1.5951 | 2E-04 |
| ST3GAL6 | 210942_s_at | ST3 beta-galactoside alpha-2,3-sialyltransferase 6 | -2.4787 | 4E-08 |
| FLT1 | 222033_s_at | fms related tyrosine kinase 1 | -1.902 | 2E-04 |
| SULF1 | 212344_at | sulfatase 1 | -2.0748 | 4E-11 |
| CMAHP | 205518_s_at | cytidine monophospho-N-acetylneuraminic acid hydroxylase, pseudogene | -1.5932 | 2E-04 |
| CTSV | 210074_at | cathepsin V | -1.8303 | 2E-04 |
| TMOD1 | 203662_s_at | tropomodulin 1 | -1.5068 | 3E-04 |
| GZMK | 206666_at | granzyme K | 1.7674 | 3E-04 |
| WFDC2 | 203892_at | WAP four-disulfide core domain 2 | 1.9199 | 3E-04 |
| ERG | 213541_s_at | ERG, ETS transcription factor | -1.5147 | 3E-04 |
| SFN | 33323_r_at | stratifin | 1.6662 | 3E-04 |
| BLNK | 207655_s_at | B-cell linker | 1.5048 | 3E-04 |
| CRHBP | 205984_at | corticotropin releasing hormone binding protein | -1.7392 | 3E-04 |
| MMP7 | 204259_at | matrix metallopeptidase 7 | 2.0864 | 3E-04 |
| PTPRD | 205712_at | protein tyrosine phosphatase, receptor type D | -1.5485 | 4E-04 |
| THSD7A | 214920_at | thrombospondin type 1 domain containing 7A | -2.265 | 7E-08 |
| CORO1A | 209083_at | coronin 1A | 1.5718 | 4E-04 |
| CXCL6 | 206336_at | C-X-C motif chemokine ligand 6 | 2.2584 | 4E-04 |
| GZMA | 205488_at | granzyme A | 1.8061 | 4E-04 |
| PODXL | 201578_at | podocalyxin like | -1.9921 | 4E-04 |
| MAP1B | 212233_at | microtubule associated protein 1B | -1.5428 | 5E-04 |
| HGF | 209960_at | hepatocyte growth factor | 1.6041 | 5E-04 |
| IL7R | 205798_at | interleukin 7 receptor | 1.5867 | 5E-04 |
| PLAT | 201860_s_at | plasminogen activator, tissue type | -2.2454 | 6E-04 |
| SST | 213921_at | somatostatin | -2.1762 | 6E-04 |
| CCL5 | 204655_at | C-C motif chemokine ligand 5 | 1.7205 | 6E-04 |
| JCHAIN | 212592_at | joining chain of multimeric IgA and IgM | 2.5427 | 7E-04 |
| CCL19 | 210072_at | C-C motif chemokine ligand 19 | 1.9313 | 8E-04 |
| IGHM | 209374_s_at | immunoglobulin heavy constant mu | 2.7773 | 8E-04 |
| ABCA8 | 204719_at | ATP binding cassette subfamily A member 8 | 1.6467 | 0.001 |
| DYNC1I1 | 205348_s_at | dynein cytoplasmic 1 intermediate chain 1 | -1.5859 | 0.001 |
| NPHS2 | 220424_at | NPHS2, podocin | -2.2605 | 0.001 |
| PTPRB | 205846_at | protein tyrosine phosphatase, receptor type B | -1.5386 | 0.001 |
| NELL1 | 206089_at | neural EGFL like 1 | -1.9668 | 0.002 |
| LTB | 207339_s_at | lymphotoxin beta | 1.7085 | 0.002 |
| IGHA2 | 217022_s_at | immunoglobulin heavy constant alpha 2 (A2m marker)///immunoglobulin heavy constant alpha 1///immunoglobulin heavy locus | 3.8071 | 0.002 |
| TRAF3IP3 | 213888_s_at | TRAF3 interacting protein 3 | 1.7581 | 0.002 |
| SFRP1 | 202036_s_at | secreted frizzled related protein 1 | 1.7806 | 0.002 |
| LYZ | 213975_s_at | lysozyme | 1.5295 | 0.003 |
| LAPTM5 | 201721_s_at | lysosomal protein transmembrane 5 | 1.642 | 0.003 |
| IGFBP1 | 205302_at | insulin like growth factor binding protein 1 | -1.925 | 0.003 |
| IL1RL1 | 207526_s_at | interleukin 1 receptor like 1 | -1.8318 | 0.003 |
| OLFM4 | 212768_s_at | olfactomedin 4 | 1.791 | 0.003 |
| LOC101930363 | 214945_at | uncharacterized LOC101930363///uncharacterized LOC101928349///uncharacterized LOC100507387///family with sequence similarity 153, member C///family with sequence similarity 153 member A///family with sequence similarity 153 member B | -2.0749 | 0.003 |
| TRBC1 | 213193_x_at | T cell receptor beta constant 1 | 1.7713 | 6E-05 |
| AKR1B10 | 206561_s_at | aldo-keto reductase family 1 member B10 | 1.7054 | 0.005 |
| IGFBP5 | 203424_s_at | insulin like growth factor binding protein 5 | -1.5514 | 0.005 |
| USP46 | 203869_at | ubiquitin specific peptidase 46 | -2.2968 | 3E-10 |
| DUSP1 | 201041_s_at | dual specificity phosphatase 1 | -1.6489 | 0.006 |
| ALOX5 | 204446_s_at | arachidonate 5-lipoxygenase | 1.6737 | 0.006 |
| MIR8071-2 | 211430_s_at | microRNA 8071-2///microRNA 8071-1///immunoglobulin heavy variable 4-31///immunoglobulin heavy constant mu///immunoglobulin heavy constant gamma 2 (G2m marker)///immunoglobulin heavy constant gamma 1 (G1m marker) | 3.3322 | 0.007 |
| REG1A | 209752_at | regenerating family member 1 alpha | 1.5169 | 0.008 |
| IGKC | 214669_x_at | immunoglobulin kappa constant | 2.0835 | 0.008 |
| IGFBP2 | 202718_at | insulin like growth factor binding protein 2 | -1.5942 | 0.009 |
| ALB | 214842_s_at | albumin | -1.7862 | 0.009 |
| VEGFA | 211527_x_at | vascular endothelial growth factor A | -1.5862 | 6E-07 |
| TAC1 | 206552_s_at | tachykinin precursor 1 | 1.6762 | 0.011 |
| IGLV1-44 | 215379_x_at | immunoglobulin lambda variable 1-44 | 2.3167 | 0.012 |
| UMOD | 206716_at | uromodulin | -1.6261 | 0.012 |
| CYAT1 | 215121_x_at | immunoglobulin lambda light chain-like///immunoglobulin lambda variable 1-44///immunoglobulin lambda constant 1 | 2.4121 | 0.014 |
| FOSB | 202768_at | FosB proto-oncogene, AP-1 transcription factor subunit | -1.8965 | 0.025 |
| SLC7A8 | 216604_s_at | solute carrier family 7 member 8 | -1.6331 | 0.029 |
| ALDOB | 214424_s_at | aldolase, fructose-bisphosphate B | -1.6128 | 0.034 |
