# Supplementary material for: Exploring the Potential Mechanism of Tang-Shen-Ning Decoction against Diabetic Nephropathy Based on the Combination of Network Pharmacology and Experimental Validation
Source: Evid Based Complement Alternat Med. 2021 Sep 9;2021:1025053. doi: 10.1155/2021/1025053 (PMC8445713; doi:10.1155/2021/1025053)
Supplement: Supplementary Materials. — Supplementary material 1: Table S1. The details of DN-DEGs in GSE30528. Supplementary material 2: Table S2. The information about topological parameters in the PPI network. Supplementary material 3: Table S3. The information of GFR and TSN-related DN-DEGs provided by Nephroseq v5. Supplementary material 4: Figure S1. Gene relative expression of TSN-related DN-DEGs in GSE30528. (A) 9 upregulated targets; (B) 14 downregulated targets. Supplementary material 5: Figure S2. Correlation between TSN-related DN-DEGs expression and GFR. [file 1025053.f1.zip › 1025053.f1/Table S2.doc]

| **The information about topological parameters in the PPI network** | | | |
| --- | --- | --- | --- |
| **gene** | **BetweennessCentrality** | **ClosenessCentrality** | **Degree** |
| CDKN1A | 0.003722702 | 0.434055118 | 61 |
| ITGA5 | 0.001415877 | 0.388888889 | 14 |
| MYO19 | 1.93E-04 | 0.375 | 20 |
| CDK2 | 0.011787244 | 0.456521739 | 100 |
| WASL | 4.88E-04 | 0.421404682 | 17 |
| CDK4 | 0.001312331 | 0.4375 | 29 |
| HNRNPA1 | 0.007421447 | 0.469648562 | 78 |
| PTPRO | 8.67E-05 | 0.404772832 | 16 |
| PTPRZ1 | 1.69E-05 | 0.347244094 | 8 |
| PTPRM | 2.00E-05 | 0.345746766 | 8 |
| PTPRK | 0.004207415 | 0.393574297 | 22 |
| PTPRG | 2.15E-05 | 0.378540773 | 13 |
| PTPRF | 2.03E-04 | 0.387521968 | 13 |
| PTPRJ | 1.79E-04 | 0.406639004 | 25 |
| AP3D1 | 7.76E-05 | 0.381487889 | 10 |
| PTPRH | 3.84E-06 | 0.347928994 | 8 |
| FOXA3 | 2.02E-04 | 0.371681416 | 6 |
| PTPRC | 0.001857551 | 0.408522464 | 38 |
| BBC3 | 1.23E-04 | 0.339230769 | 3 |
| CDC42 | 0.004457799 | 0.424650939 | 47 |
| PTPRB | 6.63E-05 | 0.389060432 | 16 |
| CDH1 | 0.00136494 | 0.409090909 | 31 |
| PTPN12 | 2.36E-04 | 0.420200095 | 22 |
| PTPN11 | 0.002145983 | 0.428363283 | 59 |
| CDC25C | 2.77E-04 | 0.413308341 | 22 |
| PTPN6 | 0.001386943 | 0.411572562 | 50 |
| PTPN4 | 2.32E-04 | 0.374363328 | 8 |
| CDK1 | 0.003369964 | 0.43880597 | 50 |
| PTPN1 | 0.003432753 | 0.42815534 | 39 |
| HMGB1 | 0.001209134 | 0.407202216 | 23 |
| CD151 | 0 | 0.262891207 | 1 |
| CACNA1I | 0 | 0.327394209 | 1 |
| FOXJ3 | 1.27E-04 | 0.371368421 | 5 |
| CD79A | 6.30E-04 | 0.372623574 | 13 |
| CD79B | 1.68E-05 | 0.34751773 | 11 |
| AP1M1 | 0.001893149 | 0.403846154 | 12 |
| DAPP1 | 2.40E-05 | 0.360588716 | 8 |
| PELP1 | 1.86E-04 | 0.414863594 | 16 |
| PHB | 0.002206857 | 0.426292895 | 38 |
| LAT | 0.002805826 | 0.414279004 | 26 |
| SLC25A3 | 2.71E-04 | 0.397118415 | 19 |
| FBXW7 | 0.001423649 | 0.422009569 | 29 |
| SYCE1 | 2.50E-05 | 0.325821943 | 3 |
| PGF | 0 | 0.300306435 | 6 |
| PGK1 | 7.53E-04 | 0.407014305 | 18 |
| SQSTM1 | 0.004435695 | 0.456521739 | 57 |
| HINT1 | 2.29E-04 | 0.327637444 | 10 |
| CFH | 1.27E-04 | 0.379354839 | 4 |
| TSC22D1 | 0.003058768 | 0.402923709 | 26 |
| WISP2 | 0 | 0.255208333 | 1 |
| RC3H1 | 0.002083891 | 0.379028792 | 25 |
| OR8D2 | 0 | 0.327394209 | 1 |
| SOCS2 | 5.64E-06 | 0.345475911 | 9 |
| HTT | 0.001584665 | 0.431718062 | 38 |
| NRP1 | 0.001284088 | 0.353790614 | 14 |
| NRP2 | 2.98E-04 | 0.306569343 | 7 |
| SMU1 | 3.91E-04 | 0.399637517 | 12 |
| RRAS2 | 0.001196594 | 0.402372263 | 18 |
| HCK | 0.002299319 | 0.422818792 | 44 |
| RPL18A | 2.76E-04 | 0.386672512 | 16 |
| SYNPO | 2.81E-04 | 0.386164623 | 22 |
| HFE2 | 0 | 0.248310811 | 1 |
| ATM | 9.43E-04 | 0.409090909 | 21 |
| CSNK2A3 | 1.73E-04 | 0.409470752 | 18 |
| U2AF1L5 | 3.72E-04 | 0.387011847 | 14 |
| HBA2 | 8.05E-04 | 0.404401651 | 19 |
| HBA1 | 8.05E-04 | 0.404401651 | 19 |
| PARK7 | 3.77E-04 | 0.395515695 | 20 |
| ASCL1 | 5.82E-05 | 0.290705339 | 4 |
| ZNF558 | 3.55E-05 | 0.332453826 | 4 |
| MPDZ | 2.94E-04 | 0.342923795 | 6 |
| ARRB1 | 0.006079608 | 0.454404946 | 71 |
| CDC45 | 1.93E-04 | 0.398373984 | 10 |
| GID8 | 6.85E-06 | 0.315 | 9 |
| PINX1 | 5.97E-04 | 0.383145091 | 10 |
| AKR1B15 | 4.61E-05 | 0.299796057 | 5 |
| ARPP19 | 4.16E-06 | 0.374363328 | 3 |
| CXCR3 | 0 | 0.281160344 | 4 |
| PLK2 | 4.72E-04 | 0.38548951 | 10 |
| AR | 0.00587991 | 0.450920245 | 79 |
| FASLG | 0.001320579 | 0.405144695 | 23 |
| ZCCHC8 | 2.57E-04 | 0.39927569 | 11 |
| GPC1 | 2.24E-04 | 0.356219709 | 4 |
| PFDN2 | 6.05E-04 | 0.384816754 | 18 |
| FAS | 0.001475471 | 0.393574297 | 22 |
| USP9X | 3.67E-04 | 0.420400381 | 23 |
| GRAP | 4.06E-05 | 0.362366475 | 11 |
| PF4V1 | 0 | 0.327394209 | 1 |
| PFDN1 | 8.57E-04 | 0.375798892 | 13 |
| AKAP17A | 1.44E-05 | 0.335361217 | 2 |
| HSPB8 | 4.38E-05 | 0.371055953 | 9 |
| IRF8 | 0.002697639 | 0.370899916 | 24 |
| PF4 | 1.63E-04 | 0.298983051 | 7 |
| CNOT11 | 3.56E-05 | 0.323432343 | 9 |
| ADAM10 | 0.001683478 | 0.366888519 | 10 |
| IARS | 3.91E-04 | 0.432141107 | 22 |
| NCOA3 | 0.002067511 | 0.425881217 | 36 |
| ACVR2B | 0.001559356 | 0.353223869 | 9 |
| PEG3 | 2.71E-04 | 0.35708502 | 7 |
| PECAM1 | 7.91E-04 | 0.370432591 | 21 |
| ACVR1 | 0.002360397 | 0.350695825 | 15 |
| TTPAL | 0 | 0.327394209 | 1 |
| ACVR2A | 3.66E-05 | 0.270137825 | 4 |
| FSD1 | 5.59E-05 | 0.388717497 | 8 |
| GADD45GIP1 | 1.48E-04 | 0.390092879 | 12 |
| PDGFRB | 0.002632216 | 0.418207681 | 46 |
| ACTB | 0.010398901 | 0.47266881 | 84 |
| HSPG2 | 5.88E-04 | 0.396404494 | 13 |
| B3GNT2 | 2.03E-05 | 0.285621762 | 3 |
| ACTA1 | 0.003079058 | 0.43902439 | 47 |
| DNAJB1 | 8.47E-04 | 0.432564983 | 28 |
| PDE4B | 1.09E-04 | 0.3828125 | 6 |
| ACP1 | 4.45E-04 | 0.402555911 | 17 |
| HSP90AB1 | 0.01115475 | 0.47649919 | 114 |
| CGRRF1 | 0 | 0.331953331 | 1 |
| HSP90AA1 | 0.021630903 | 0.498868778 | 136 |
| SMURF2 | 0.001675454 | 0.394984326 | 24 |
| HSPB2 | 7.36E-04 | 0.392 | 18 |
| CEACAM21 | 2.33E-04 | 0.336256195 | 4 |
| HSPA9 | 0.002716387 | 0.456758156 | 63 |
| KHDRBS1 | 0.001589144 | 0.43045388 | 44 |
| HSPA8 | 0.005091254 | 0.472922252 | 94 |
| HSPB1 | 0.004678444 | 0.450689831 | 59 |
| HSPA4 | 0.005457544 | 0.460093897 | 71 |
| HSPA1B | 0.003717935 | 0.461055933 | 66 |
| HSPA1A | 0.003717935 | 0.461055933 | 66 |
| LIME1 | 1.37E-06 | 0.354501608 | 4 |
| HSPA1L | 8.09E-04 | 0.435340573 | 39 |
| PCNA | 0.00147878 | 0.422818792 | 45 |
| HSD17B4 | 9.29E-05 | 0.410805776 | 13 |
| SERPINA5 | 3.88E-04 | 0.34439672 | 10 |
| FXR1 | 6.07E-04 | 0.402188782 | 21 |
| LAMA1 | 6.74E-04 | 0.375638842 | 11 |
| PALB2 | 9.18E-04 | 0.376762067 | 7 |
| CDKAL1 | 6.95E-06 | 0.326062847 | 2 |
| LAX1 | 8.99E-05 | 0.361920394 | 7 |
| GDPD1 | 0.001108652 | 0.355501814 | 20 |
| PCDH1 | 2.03E-05 | 0.351394422 | 2 |
| COL7A1 | 1.48E-04 | 0.378865979 | 6 |
| SGO2 | 0 | 0.327394209 | 3 |
| HPX | 3.77E-04 | 0.337931034 | 4 |
| STAMBP | 5.32E-04 | 0.388375165 | 14 |
| HRAS | 0.002268609 | 0.419400856 | 32 |
| LRRK1 | 4.03E-04 | 0.387351779 | 18 |
| HPRT1 | 8.71E-05 | 0.379844961 | 11 |
| HPGD | 0 | 0.344665885 | 1 |
| QTRT2 | 0 | 0.327394209 | 1 |
| COL2A1 | 0.001133994 | 0.35665184 | 9 |
| CUBN | 2.68E-04 | 0.375 | 8 |
| HP | 5.58E-04 | 0.387011847 | 11 |
| PARK2 | 0.008411265 | 0.475471698 | 80 |
| CDC42EP3 | 2.41E-05 | 0.348478862 | 6 |
| PAK3 | 2.06E-05 | 0.361031519 | 8 |
| PAK2 | 8.88E-04 | 0.429615197 | 34 |
| SERPINE1 | 5.22E-04 | 0.34765471 | 15 |
| PLK3 | 8.56E-05 | 0.375638842 | 8 |
| PRDX1 | 5.58E-04 | 0.426911907 | 33 |
| P4HB | 0.00232998 | 0.419600381 | 31 |
| ACKR2 | 7.02E-05 | 0.350695825 | 2 |
| CCR3 | 4.55E-05 | 0.319333816 | 5 |
| CCR1 | 1.81E-04 | 0.338190184 | 8 |
| CCR5 | 6.81E-04 | 0.376762067 | 14 |
| CCR4 | 1.53E-05 | 0.310782241 | 2 |
| CLTC | 0.002667135 | 0.45746888 | 63 |
| MAEA | 2.19E-04 | 0.343859649 | 8 |
| CNBP | 1.17E-05 | 0.345611285 | 5 |
| ORM1 | 2.86E-04 | 0.346970889 | 5 |
| DOK2 | 6.06E-05 | 0.376279863 | 14 |
| CLU | 0.004311669 | 0.421404682 | 33 |
| OR3A2 | 0 | 0.327394209 | 1 |
| ENC1 | 4.08E-06 | 0.380008617 | 8 |
| IMMT | 0.002077348 | 0.418207681 | 28 |
| GREM2 | 0 | 0.248310811 | 1 |
| GCN1 | 4.59E-04 | 0.427532719 | 19 |
| RANBP3 | 5.68E-04 | 0.390784227 | 8 |
| COPS5 | 0.008786003 | 0.460093897 | 99 |
| CHST8 | 0 | 0.24740533 | 1 |
| COPS6 | 0.001856361 | 0.419400856 | 44 |
| BRCA1 | 0.006968289 | 0.445679636 | 70 |
| YWHAQ | 0.005951465 | 0.4659271 | 84 |
| CKAP4 | 0.001583355 | 0.401822323 | 23 |
| BMPR2 | 0.001177065 | 0.359853121 | 12 |
| PTK2 | 0.003694872 | 0.443438914 | 57 |
| BMPR1B | 0.003505525 | 0.388888889 | 18 |
| BMPR1A | 0.004151863 | 0.384648932 | 22 |
| CUL3 | 0.015829765 | 0.472162741 | 128 |
| BMP2 | 0.020207911 | 0.330213403 | 29 |
| CUL4B | 0.001799023 | 0.435555556 | 54 |
| CXCR5 | 0 | 0.28089172 | 3 |
| GJA3 | 2.21E-05 | 0.345205479 | 5 |
| PRDX3 | 2.65E-04 | 0.405144695 | 19 |
| GJA5 | 2.11E-05 | 0.327637444 | 4 |
| PTCH1 | 9.42E-04 | 0.370277078 | 11 |
| GJA1 | 0.010841679 | 0.393223362 | 41 |
| P3H1 | 1.19E-04 | 0.388888889 | 8 |
| CFB | 5.84E-05 | 0.36416185 | 7 |
| LRP1B | 2.84E-04 | 0.368421053 | 7 |
| PSMD4 | 0.002762137 | 0.449770525 | 35 |
| MYLK3 | 5.14E-05 | 0.370121695 | 3 |
| GADD45G | 0.001243389 | 0.393574297 | 26 |
| CABLES1 | 1.90E-05 | 0.359853121 | 2 |
| BCAR3 | 3.59E-04 | 0.383478261 | 12 |
| PSMD2 | 0.002472333 | 0.418604651 | 40 |
| PSMC3 | 5.68E-04 | 0.415841584 | 25 |
| DNAAF2 | 5.10E-05 | 0.377406932 | 8 |
| BCL3 | 2.64E-05 | 0.382978723 | 11 |
| GFAP | 0.00103509 | 0.428571429 | 23 |
| KLK3 | 5.48E-04 | 0.379191745 | 9 |
| LYVE1 | 0 | 0.275366844 | 1 |
| APOE | 0.001416745 | 0.387011847 | 17 |
| APP | 0.079651482 | 0.525625745 | 173 |
| APOC3 | 1.53E-04 | 0.331953331 | 3 |
| APOC2 | 2.40E-04 | 0.345746766 | 7 |
| APOC1 | 0 | 0.329596413 | 5 |
| PSMA3 | 0.003797983 | 0.445005045 | 48 |
| APOA2 | 3.95E-05 | 0.334597876 | 10 |
| APOA1 | 0.003785701 | 0.40347667 | 28 |
| MAD1L1 | 1.30E-04 | 0.362366475 | 7 |
| APOA4 | 0 | 0.329104478 | 4 |
| CNOT6L | 1.13E-05 | 0.322015334 | 10 |
| SGSM3 | 1.22E-04 | 0.373254338 | 5 |
| APLP1 | 1.38E-04 | 0.380664653 | 13 |
| PSAP | 2.30E-04 | 0.376923077 | 14 |
| APC | 0.003128614 | 0.412149533 | 36 |
| GBA | 2.24E-04 | 0.39927569 | 13 |
| KLK6 | 2.24E-04 | 0.376440461 | 5 |
| PRPF19 | 5.78E-04 | 0.422616195 | 30 |
| YWHAH | 0.003057836 | 0.444556452 | 43 |
| ZAP70 | 0.001717929 | 0.420400381 | 48 |
| YWHAB | 0.002327413 | 0.457231726 | 61 |
| PRSS1 | 7.19E-04 | 0.418009479 | 8 |
| CEP295 | 3.34E-05 | 0.334724858 | 2 |
| ARHGAP17 | 2.70E-04 | 0.40871177 | 11 |
| YWHAG | 0.008702846 | 0.477789816 | 84 |
| PRSS3 | 4.92E-04 | 0.401822323 | 10 |
| YWHAE | 0.008967511 | 0.464944649 | 73 |
| ANGPTL3 | 3.63E-05 | 0.333459357 | 3 |
| TNKS1BP1 | 6.91E-04 | 0.376119403 | 14 |
| ANXA6 | 2.65E-04 | 0.394454383 | 11 |
| YES1 | 0.002725124 | 0.423224568 | 46 |
| GAPDH | 0.00330629 | 0.461780105 | 68 |
| ANXA2 | 0.002609028 | 0.451381781 | 48 |
| STUB1 | 0.004009228 | 0.457231726 | 64 |
| DOCK7 | 2.31E-04 | 0.39927569 | 16 |
| MAP2K7 | 8.08E-05 | 0.367806505 | 13 |
| WT1 | 7.55E-05 | 0.351814918 | 4 |
| NR5A2 | 2.57E-05 | 0.346834448 | 5 |
| SRRM1 | 0.001080587 | 0.414668547 | 27 |
| AMPD3 | 0 | 0.327394209 | 1 |
| MAPK7 | 6.04E-04 | 0.411380597 | 23 |
| SUSD4 | 0 | 0.24740533 | 1 |
| MAPK8 | 0.003870034 | 0.447488584 | 52 |
| MAPK1 | 0.008471068 | 0.466419884 | 79 |
| MAPK3 | 0.005312874 | 0.454639175 | 60 |
| AMBP | 4.59E-04 | 0.39392586 | 11 |
| SIT1 | 1.32E-06 | 0.357809331 | 9 |
| PRKCQ | 2.11E-04 | 0.389748122 | 18 |
| PRKCG | 9.75E-04 | 0.399637517 | 23 |
| DERL1 | 3.98E-04 | 0.407578558 | 10 |
| ALOX5AP | 0 | 0.283601286 | 4 |
| PRKCE | 7.11E-04 | 0.423021583 | 28 |
| PRKCA | 0.004587745 | 0.44077961 | 57 |
| USP47 | 7.96E-05 | 0.369811321 | 5 |
| WAS | 4.51E-04 | 0.395515695 | 28 |
| GTF2F2 | 3.17E-04 | 0.39061116 | 12 |
| ALOX5 | 0.010667376 | 0.395515695 | 23 |
| PRKCD | 0.003325389 | 0.450689831 | 52 |
| PRKCB | 0.001186349 | 0.416627303 | 31 |
| VTN | 0.001223935 | 0.392523364 | 16 |
| PRKACA | 0.006623304 | 0.437717122 | 53 |
| PATJ | 3.40E-04 | 0.35665184 | 8 |
| VLDLR | 1.52E-05 | 0.313432836 | 3 |
| EZR | 0.002276789 | 0.430243902 | 35 |
| SRGN | 0.001005813 | 0.36147541 | 6 |
| ALB | 0.132952 | 0.486486486 | 182 |
| VIM | 0.003365693 | 0.45393721 | 55 |
| AKT1 | 0.00563662 | 0.461780105 | 72 |
| VEGFB | 6.95E-05 | 0.299287411 | 6 |
| VHL | 0.003685989 | 0.438369781 | 57 |
| GSK3B | 0.005001248 | 0.450229709 | 65 |
| PRB3 | 0 | 0.327394209 | 1 |
| VEGFA | 0.012261023 | 0.379844961 | 31 |
| GSN | 0.002123244 | 0.447943118 | 35 |
| VDAC1 | 0.001589294 | 0.418406072 | 34 |
| SACS | 4.94E-05 | 0.366583541 | 5 |
| AHSG | 0.002338522 | 0.396226415 | 10 |
| VDAC2 | 0.001014103 | 0.418406072 | 21 |
| SORBS3 | 0.001481026 | 0.390092879 | 16 |
| VAV1 | 0.001109309 | 0.410614525 | 44 |
| PRRG4 | 0 | 0.275366844 | 1 |
| TSPAN17 | 9.80E-05 | 0.306462821 | 3 |
| AGA | 1.56E-06 | 0.336384439 | 6 |
| SLC25A13 | 3.00E-04 | 0.421807747 | 11 |
| NR3C1 | 0.001644717 | 0.4375 | 49 |
| USP1 | 1.18E-04 | 0.368729097 | 14 |
| FBXO25 | 0.001376022 | 0.418207681 | 27 |
| ATP6AP2 | 2.23E-04 | 0.351394422 | 13 |
| UQCRC2 | 3.71E-04 | 0.40871177 | 18 |
| GRIN1 | 4.41E-04 | 0.375638842 | 17 |
| AP1B1 | 1.93E-04 | 0.406264394 | 14 |
| PPBP | 0 | 0.330460847 | 4 |
| AJUBA | 4.18E-05 | 0.373096447 | 8 |
| UIMC1 | 4.44E-04 | 0.394984326 | 12 |
| PPARG | 0.001119569 | 0.431718062 | 37 |
| ZACN | 5.35E-05 | 0.325821943 | 4 |
| GRK2 | 0.001994025 | 0.417613636 | 24 |
| GID4 | 2.57E-07 | 0.277795276 | 3 |
| G3BP1 | 0.001057437 | 0.440559441 | 34 |
| GRB2 | 0.039672573 | 0.5 | 159 |
| PPARD | 1.47E-04 | 0.376923077 | 14 |
| PPARA | 2.82E-04 | 0.385995624 | 16 |
| ADRA1B | 1.02E-04 | 0.360146999 | 4 |
| GRK6 | 9.21E-05 | 0.376119403 | 9 |
| GPS1 | 2.17E-04 | 0.383311604 | 15 |
| PON2 | 0.001827659 | 0.372151899 | 7 |
| PTPN22 | 1.07E-04 | 0.390438247 | 16 |
| GRK5 | 0.001770371 | 0.408144378 | 34 |
| ADH1C | 0 | 0.252360515 | 3 |
| ADH1B | 0.002373152 | 0.337413925 | 11 |
| UBE3A | 0.001491232 | 0.432989691 | 39 |
| POLR2L | 2.28E-04 | 0.377406932 | 10 |
| PON1 | 2.32E-05 | 0.340672074 | 3 |
| SPATA31A7 | 5.36E-05 | 0.340935446 | 2 |
| POLR2H | 2.30E-04 | 0.39061116 | 15 |
| LUC7L2 | 0.001792561 | 0.435125802 | 28 |
| ACTR2 | 0.007398638 | 0.419600381 | 30 |
| UBB | 0.001403502 | 0.445679636 | 42 |
| SEPTIN4 | 3.88E-06 | 0.351814918 | 7 |
| ACACA | 0.001013527 | 0.402372263 | 16 |
| U2AF1 | 3.72E-04 | 0.387011847 | 14 |
| UBE2Z | 7.62E-05 | 0.325341203 | 4 |
| RANBP9 | 0.006667749 | 0.438152012 | 48 |
| SRRT | 3.66E-04 | 0.413114754 | 18 |
| PML | 0.002363168 | 0.424446583 | 42 |
| TUBG1 | 0.002889557 | 0.446356275 | 52 |
| SERPINA3 | 6.45E-04 | 0.363861386 | 10 |
| A1BG | 1.56E-04 | 0.35665184 | 4 |
| TTR | 0.001093009 | 0.408901252 | 20 |
| A2M | 0.002352749 | 0.409090909 | 25 |
| TMEM25 | 4.84E-04 | 0.355072464 | 7 |
| HAVCR2 | 9.35E-06 | 0.372151899 | 5 |
| SH2B3 | 1.67E-05 | 0.372623574 | 11 |
| ACKR4 | 3.51E-05 | 0.325221239 | 2 |
| PLK1 | 0.002969813 | 0.442548921 | 40 |
| PLG | 0.005231084 | 0.391825855 | 30 |
| PLCG2 | 4.85E-04 | 0.40347667 | 34 |
| PINK1 | 1.95E-04 | 0.401822323 | 23 |
| PLCG1 | 0.00213886 | 0.43045388 | 61 |
| RABAC1 | 1.69E-04 | 0.365217391 | 13 |
| PLD2 | 2.34E-04 | 0.400909091 | 18 |
| PLD1 | 2.59E-04 | 0.393574297 | 15 |
| BAG6 | 0.009043848 | 0.435340573 | 57 |
| PLAU | 2.72E-04 | 0.323313783 | 11 |
| PLAT | 0.007549031 | 0.385658067 | 34 |
| PLCB2 | 0 | 0.331953331 | 3 |
| PLA2G4F | 0 | 0.327394209 | 1 |
| PLAG1 | 2.03E-05 | 0.351394422 | 2 |
| CHMP5 | 6.65E-05 | 0.365368683 | 7 |
| LAMTOR5 | 0.002354118 | 0.397118415 | 17 |
| GLRX3 | 7.89E-04 | 0.374522293 | 11 |
| RAB7A | 0.001143741 | 0.40403115 | 29 |
| LIMA1 | 0.001246403 | 0.439900249 | 41 |
| OR2T6 | 0 | 0.327394209 | 1 |
| PI4KA | 1.62E-04 | 0.390265487 | 9 |
| PIN1 | 0.004387809 | 0.436849926 | 51 |
| TUBA1A | 0.001726277 | 0.444108761 | 50 |
| PIK3R1 | 0.006792697 | 0.453703704 | 88 |
| CDCP1 | 3.59E-04 | 0.383979103 | 6 |
| PIK3CA | 9.53E-04 | 0.415645617 | 27 |
| PIGR | 1.21E-04 | 0.319796954 | 6 |
| FMR1 | 3.81E-04 | 0.392 | 17 |
| FN1 | 0.034818497 | 0.495505618 | 129 |
| SERPINI1 | 0 | 0.292828685 | 2 |
| LRP8 | 4.31E-04 | 0.364915184 | 8 |
| ASB15 | 4.88E-04 | 0.421404682 | 17 |
| FLT1 | 0.003529642 | 0.382978723 | 27 |
| GOLT1B | 2.76E-04 | 0.360588716 | 16 |
| SERPINA1 | 6.70E-04 | 0.356796117 | 15 |
| FLNA | 0.00606703 | 0.45746888 | 65 |
| RMND5A | 0.001123696 | 0.362366475 | 10 |
| ZNF232 | 2.22E-05 | 0.341728012 | 2 |
| LMF1 | 1.51E-04 | 0.28776509 | 3 |
| NTRK1 | 0.052771501 | 0.510416667 | 213 |
| NT5E | 7.11E-05 | 0.355788624 | 4 |
| FKBP4 | 7.66E-04 | 0.411380597 | 20 |
| HAX1 | 2.84E-04 | 0.413114754 | 19 |
| YBX1 | 0.002829543 | 0.435340573 | 51 |
| TRPV4 | 4.19E-06 | 0.350695825 | 8 |
| CDC42EP2 | 5.25E-06 | 0.342790517 | 2 |
| FGR | 0.001416789 | 0.415254237 | 27 |
| FAM174A | 0 | 0.318641618 | 1 |
| FGL1 | 5.54E-05 | 0.311881188 | 5 |
| FGFR3 | 2.58E-04 | 0.379028792 | 18 |
| ZBTB17 | 1.09E-04 | 0.372623574 | 12 |
| FGFR2 | 2.55E-04 | 0.398914518 | 18 |
| COL25A1 | 0 | 0.36857501 | 4 |
| NPHS1 | 3.24E-04 | 0.381322957 | 8 |
| MAP1LC3A | 3.67E-04 | 0.413308341 | 19 |
| NOV | 7.25E-04 | 0.377892031 | 11 |
| FBLN1 | 0.001430848 | 0.389403974 | 16 |
| GAR1 | 2.16E-04 | 0.380008617 | 7 |
| FANCB | 0 | 0.25257732 | 1 |
| NOTCH1 | 0.001802568 | 0.424855491 | 34 |
| SIAE | 7.12E-05 | 0.287859008 | 3 |
| RACK1 | 0.00388719 | 0.436417615 | 53 |
| CNOT2 | 2.22E-04 | 0.34001542 | 15 |
| TERF2IP | 0.002492748 | 0.370899916 | 16 |
| PTK2B | 0.001048071 | 0.43003413 | 40 |
| CNOT3 | 3.37E-04 | 0.362515413 | 11 |
| NOS2 | 0.001505135 | 0.402188782 | 30 |
| LZTS2 | 5.86E-04 | 0.374363328 | 11 |
| NME2 | 7.36E-04 | 0.373254338 | 21 |
| SCAF1 | 6.19E-05 | 0.349722443 | 3 |
| TUBA1B | 0.001373893 | 0.428988327 | 34 |
| CYCS | 2.09E-04 | 0.381818182 | 13 |
| NUFIP1 | 2.00E-04 | 0.371212121 | 14 |
| TUB | 5.30E-07 | 0.360736196 | 5 |
| TTN | 0.001858434 | 0.431718062 | 26 |
| NLRC4 | 0 | 0.327394209 | 3 |
| NGF | 2.13E-04 | 0.369037657 | 8 |
| TSNAX | 0.001524672 | 0.377730193 | 23 |
| NFKBIB | 2.35E-04 | 0.392348754 | 24 |
| NFKB1 | 0.00212791 | 0.424855491 | 54 |
| TSG101 | 0.001330661 | 0.416824197 | 25 |
| NFKBIA | 0.001282003 | 0.427740058 | 43 |
| GABBR1 | 2.29E-04 | 0.385321101 | 5 |
| GAB1 | 4.26E-04 | 0.412149533 | 37 |
| FYN | 0.014556298 | 0.461055933 | 110 |
| ABI1 | 1.40E-04 | 0.382646421 | 25 |
| ACKR1 | 0 | 0.284607938 | 2 |
| PCBP3 | 0 | 0.255578093 | 1 |
| GREM1 | 3.09E-04 | 0.323669725 | 3 |
| FUS | 0.005094357 | 0.456758156 | 59 |
| POTEF | 1.48E-05 | 0.290609555 | 2 |
| NEDD9 | 6.39E-04 | 0.405890474 | 26 |
| TRIP6 | 0.002576632 | 0.39480752 | 26 |
| SEPTIN2 | 4.88E-04 | 0.406077348 | 13 |
| LOC101930123 | 4.20E-04 | 0.383645063 | 10 |
| NEDD4 | 0.004965238 | 0.445454545 | 51 |
| F10 | 1.31E-04 | 0.31488754 | 8 |
| THRAP3 | 0.001779425 | 0.425881217 | 27 |
| F3 | 0.003009348 | 0.337801609 | 13 |
| IGDCC4 | 0 | 0.327394209 | 1 |
| F7 | 9.62E-04 | 0.362366475 | 9 |
| F2 | 0.001278254 | 0.377245509 | 17 |
| TRAF6 | 0.009036657 | 0.467408585 | 88 |
| F2RL1 | 1.62E-04 | 0.350695825 | 5 |
| PPIL3 | 5.43E-05 | 0.358245329 | 4 |
| NDUFB6 | 1.04E-04 | 0.369192131 | 3 |
| TIMM13 | 1.07E-04 | 0.37388724 | 6 |
| COX3 | 2.35E-05 | 0.342125679 | 4 |
| ETV6 | 1.27E-04 | 0.372151899 | 12 |
| USP37 | 5.77E-04 | 0.394454383 | 16 |
| COX1 | 3.27E-04 | 0.345611285 | 4 |
| TP53 | 0.019831724 | 0.477789816 | 136 |
| PAN2 | 0.004085862 | 0.434055118 | 51 |
| RNF10 | 6.32E-06 | 0.384648932 | 5 |
| ETF1 | 5.20E-04 | 0.39480752 | 9 |
| ESR2 | 3.02E-04 | 0.404772832 | 23 |
| TNXB | 0 | 0.275452842 | 2 |
| ESR1 | 0.011475889 | 0.474703983 | 112 |
| TOP1 | 2.13E-04 | 0.403846154 | 19 |
| TUBB | 0.004291321 | 0.4704 | 80 |
| ERBB4 | 4.25E-04 | 0.398373984 | 22 |
| CLEC3B | 1.54E-04 | 0.369656329 | 10 |
| EPS15 | 0.002109199 | 0.436849926 | 45 |
| CLDN5 | 9.89E-06 | 0.299084435 | 5 |
| SLA2 | 5.27E-04 | 0.385321101 | 8 |
| GABARAPL1 | 3.15E-05 | 0.369346734 | 6 |
| ASCC2 | 1.49E-04 | 0.373412362 | 8 |
| FOXP3 | 3.73E-04 | 0.369966443 | 8 |
| EPHB2 | 3.49E-04 | 0.367806505 | 13 |
| TRAT1 | 4.81E-05 | 0.369966443 | 12 |
| DMTN | 2.04E-05 | 0.385995624 | 7 |
| STX12 | 3.68E-04 | 0.337931034 | 7 |
| ENSA | 1.21E-05 | 0.338449731 | 2 |
| TK1 | 0.002760431 | 0.425060241 | 31 |
| GAB2 | 3.40E-04 | 0.407955597 | 29 |
| EDC4 | 2.54E-04 | 0.379191745 | 8 |
| TJP1 | 0.002485582 | 0.404587156 | 26 |
| ENG | 4.79E-04 | 0.310235667 | 10 |
| TIAM1 | 0.001282873 | 0.403107861 | 18 |
| LCMT2 | 5.23E-04 | 0.364764268 | 3 |
| ELN | 2.95E-05 | 0.320843943 | 4 |
| EMD | 0.001750813 | 0.419800095 | 33 |
| ELK1 | 7.52E-05 | 0.408901252 | 12 |
| TH | 5.86E-05 | 0.377084224 | 9 |
| THBS1 | 0.001312592 | 0.371524853 | 19 |
| TGM1 | 1.05E-04 | 0.379518072 | 6 |
| BCAN | 3.19E-05 | 0.271301138 | 3 |
| TRAPPC11 | 5.26E-05 | 0.38381201 | 5 |
| DEF6 | 2.81E-05 | 0.375 | 6 |
| TGFB3 | 4.26E-05 | 0.297370196 | 4 |
| TGFB2 | 0.001679157 | 0.361771944 | 10 |
| LOC100996724 | 3.21E-04 | 0.372938689 | 11 |
| TGFB1 | 0.010875015 | 0.41253508 | 29 |
| TFPI | 6.68E-04 | 0.358390898 | 8 |
| GPIHBP1 | 0 | 0.277795276 | 1 |
| TFRC | 0.001813985 | 0.432989691 | 23 |
| MPV17 | 1.25E-04 | 0.344800625 | 5 |
| EIF4A1 | 1.08E-04 | 0.402372263 | 15 |
| WRAP73 | 5.25E-04 | 0.362366475 | 17 |
| EGFR | 0.041744431 | 0.513686663 | 182 |
| CMBL | 3.64E-05 | 0.367041199 | 5 |
| UBC | 0.019453595 | 0.484615385 | 112 |
| MMP9 | 9.40E-04 | 0.358828316 | 14 |
| TFCP2 | 1.32E-04 | 0.361327325 | 10 |
| TF | 0.006635726 | 0.39113082 | 23 |
| MMP7 | 0.007409738 | 0.35650768 | 36 |
| TERF2 | 0.001175696 | 0.383311604 | 27 |
| MMP1 | 7.15E-04 | 0.333207405 | 11 |
| KIAA1191 | 1.97E-04 | 0.386333771 | 7 |
| SH3KBP1 | 0.002384481 | 0.427740058 | 51 |
| DPP4 | 5.55E-05 | 0.328247116 | 6 |
| MKLN1 | 1.33E-04 | 0.336898396 | 10 |
| DOK1 | 3.67E-04 | 0.390957447 | 29 |
| GJC2 | 0 | 0.327394209 | 1 |
| MID1 | 1.17E-04 | 0.364764268 | 12 |
| KCNK16 | 0 | 0.282330346 | 1 |
| SMURF1 | 0.005460647 | 0.443885254 | 58 |
| OBP2A | 1.10E-04 | 0.289275172 | 2 |
| SKIV2L2 | 3.39E-04 | 0.400726942 | 17 |
| NOS1AP | 4.03E-04 | 0.396404494 | 10 |
| MIA2 | 1.08E-04 | 0.378054008 | 5 |
| MGP | 0 | 0.248310811 | 1 |
| FGA | 6.59E-04 | 0.384816754 | 12 |
| PDZRN4 | 0 | 0.327394209 | 1 |
| HERPUD1 | 1.50E-04 | 0.386842105 | 8 |
| HIST2H3C | 9.72E-05 | 0.396048496 | 11 |
| MET | 0.00262286 | 0.420801527 | 57 |
| CEP57 | 6.23E-04 | 0.395338413 | 21 |
| NUTM1 | 0 | 0.283510125 | 1 |
| MOXD1 | 0.008003717 | 0.328614009 | 15 |
| RAB8A | 5.03E-04 | 0.370121695 | 12 |
| MAP3K5 | 8.85E-04 | 0.413308341 | 37 |
| MEOX1 | 1.48E-04 | 0.362366475 | 7 |
| TLN2 | 0 | 0.332453826 | 2 |
| FCGRT | 0 | 0.327394209 | 3 |
| PARP11 | 0 | 0.290418176 | 3 |
| FCGR3A | 1.58E-04 | 0.377084224 | 10 |
| MAP3K4 | 7.32E-04 | 0.383645063 | 20 |
| MEF2D | 0.001946916 | 0.39269813 | 11 |
| DYNC1H1 | 0.002579352 | 0.445679636 | 37 |
| HNF1A | 0.001067873 | 0.400544959 | 14 |
| HSPB6 | 7.84E-06 | 0.358828316 | 5 |
| DMWD | 5.93E-04 | 0.381818182 | 9 |
| IQCB1 | 0.001763563 | 0.421807747 | 42 |
| KIAA1429 | 5.95E-05 | 0.378865979 | 8 |
| PDE4DIP | 3.21E-04 | 0.372938689 | 11 |
| DMD | 3.19E-04 | 0.40347667 | 12 |
| TMEM17 | 0.003334123 | 0.387351779 | 28 |
| AKR1B10 | 0.005388216 | 0.343190661 | 12 |
| TAZ | 0.001146599 | 0.341860465 | 9 |
| DLG1 | 4.32E-04 | 0.39061116 | 15 |
| NME1-NME2 | 7.36E-04 | 0.373254338 | 21 |
| SOSTDC1 | 0 | 0.248310811 | 3 |
| CLCA2 | 0 | 0.327394209 | 1 |
| LCP2 | 3.60E-04 | 0.408901252 | 26 |
| SNCAIP | 8.68E-05 | 0.372151899 | 14 |
| LCK | 0.031873515 | 0.467408585 | 153 |
| DHCR24 | 1.98E-05 | 0.378216123 | 5 |
| TAF7 | 7.56E-06 | 0.365217391 | 6 |
| POT1 | 0.003303572 | 0.395515695 | 28 |
| LCN1 | 3.16E-05 | 0.344935471 | 4 |
| DGUOK | 6.95E-05 | 0.381322957 | 11 |
| NDUFA7 | 1.18E-04 | 0.331703648 | 7 |
| CNOT10 | 3.68E-04 | 0.343190661 | 11 |
| NCBP1 | 7.74E-04 | 0.406826568 | 20 |
| CFD | 0 | 0.327394209 | 3 |
| RGMA | 1.63E-04 | 0.28 | 2 |
| SYK | 0.003197297 | 0.445679636 | 62 |
| CHRDL2 | 0 | 0.248310811 | 1 |
| SYN1 | 8.11E-04 | 0.41369606 | 25 |
| HERC5 | 1.04E-04 | 0.380993521 | 11 |
| BCAR1 | 5.67E-04 | 0.405330882 | 29 |
| ARMC8 | 2.87E-04 | 0.368883312 | 7 |
| MED25 | 2.92E-05 | 0.362962963 | 8 |
| HIST2H3D | 9.72E-05 | 0.396048496 | 11 |
| GADD45A | 5.81E-04 | 0.394101877 | 26 |
| SRI | 1.94E-04 | 0.381322957 | 8 |
| AKR1D1 | 0 | 0.255578093 | 1 |
| DDB1 | 0.002548733 | 0.437934459 | 36 |
| BDH2 | 0 | 0.331953331 | 3 |
| SRC | 0.018123719 | 0.488913525 | 133 |
| UBQLN4 | 0.002431493 | 0.386842105 | 24 |
| MYL4 | 0 | 0.358245329 | 2 |
| DCC | 4.56E-04 | 0.381652964 | 9 |
| DCN | 0.001333625 | 0.391825855 | 13 |
| SPTBN1 | 0.001486749 | 0.44077961 | 33 |
| SDF4 | 8.52E-05 | 0.389920424 | 9 |
| DBN1 | 0.001311366 | 0.427947598 | 37 |
| LOC653513 | 3.21E-04 | 0.372938689 | 11 |
| COPS4 | 5.00E-04 | 0.39269813 | 20 |
| CDC42SE1 | 5.25E-06 | 0.342790517 | 2 |
| MYH9 | 0.003883972 | 0.452772074 | 59 |
| BAG4 | 2.70E-04 | 0.376119403 | 12 |
| SPP1 | 0.001216453 | 0.389748122 | 21 |
| BAG5 | 2.09E-04 | 0.389060432 | 14 |
| ANGPTL4 | 1.81E-04 | 0.36416185 | 7 |
| GADD45B | 9.19E-05 | 0.352377147 | 14 |
| THY1 | 1.60E-07 | 0.332453826 | 4 |
| DGKG | 2.09E-05 | 0.353082466 | 2 |
| DAD1 | 3.00E-05 | 0.316129032 | 2 |
| CD55 | 5.45E-05 | 0.333711691 | 5 |
| FXR2 | 6.15E-04 | 0.4 | 17 |
| SPI1 | 4.46E-05 | 0.35435918 | 8 |
| SPARC | 3.74E-04 | 0.353932584 | 10 |
| SPAST | 0 | 0.327394209 | 1 |
| RANBP10 | 7.93E-04 | 0.348341232 | 11 |
| SOX4 | 2.31E-04 | 0.337284895 | 4 |
| MUC1 | 1.25E-04 | 0.397476341 | 19 |
| SOS1 | 6.11E-04 | 0.418406072 | 33 |
| SOD1 | 0.00243939 | 0.403107861 | 26 |
| CCDC8 | 0.008775685 | 0.462022001 | 77 |
| AP4E1 | 3.03E-04 | 0.346289753 | 4 |
| CD48 | 4.84E-06 | 0.343457944 | 5 |
| CRB1 | 1.31E-04 | 0.34001542 | 3 |
| CD44 | 0.004257591 | 0.416430595 | 33 |
| UBIAD1 | 2.77E-05 | 0.294294294 | 2 |
| SNCA | 0.084668018 | 0.496621622 | 212 |
| CD38 | 1.77E-05 | 0.34399376 | 8 |
| EEF2K | 4.20E-04 | 0.383645063 | 10 |
| DICER1 | 8.24E-04 | 0.393048128 | 11 |
| CEP126 | 3.02E-04 | 0.352236422 | 13 |
| SNCB | 3.87E-04 | 0.387351779 | 15 |
| KIAA0232 | 5.95E-05 | 0.35464415 | 3 |
| COTL1 | 1.52E-04 | 0.358099878 | 7 |
| TM9SF4 | 1.08E-04 | 0.311770944 | 4 |
| CNOT7 | 0.001614598 | 0.382480486 | 18 |
| CD28 | 2.87E-05 | 0.365975104 | 12 |
| LAMP2 | 4.83E-05 | 0.379354839 | 12 |
| EFTUD2 | 7.23E-04 | 0.423224568 | 33 |
| MS4A1 | 9.37E-06 | 0.343057176 | 4 |
| CNOT8 | 1.19E-04 | 0.338190184 | 10 |
| LAMA3 | 1.33E-04 | 0.339230769 | 6 |
| EEF1A2 | 8.44E-04 | 0.402555911 | 25 |
| EEF1A1 | 0.004957592 | 0.461780105 | 78 |
| OBSL1 | 0.005891531 | 0.449770525 | 73 |
| COPS2 | 3.34E-04 | 0.3828125 | 21 |
| NEDD1 | 6.04E-04 | 0.366583541 | 17 |
| KRT16 | 2.78E-04 | 0.415841584 | 17 |
| USP25 | 5.40E-04 | 0.389748122 | 11 |
| KRT10 | 5.35E-04 | 0.428988327 | 24 |
| KRT14 | 2.45E-04 | 0.410423453 | 16 |
| KRT13 | 4.65E-04 | 0.391651865 | 14 |
| GPR52 | 5.12E-05 | 0.307960894 | 3 |
| TUBB1 | 1.59E-04 | 0.387351779 | 14 |
| KRT6B | 1.31E-04 | 0.394630872 | 10 |
| KRT9 | 5.20E-04 | 0.423427748 | 21 |
| KRT8 | 6.01E-04 | 0.410997204 | 23 |
| SAP30BP | 4.21E-05 | 0.384481255 | 8 |
| KRT6A | 2.39E-04 | 0.405890474 | 14 |
| KRT5 | 5.05E-04 | 0.424855491 | 25 |
| KRAS | 0.001061689 | 0.396404494 | 19 |
| NEDD4L | 0.001980982 | 0.416824197 | 33 |
| E2F1 | 0.00105819 | 0.397476341 | 24 |
| KRT1 | 7.21E-04 | 0.427118644 | 26 |
| DYRK1A | 5.41E-04 | 0.407955597 | 18 |
| TOR1A | 1.71E-05 | 0.346153846 | 5 |
| LRRK2 | 0.001778811 | 0.420400381 | 47 |
| CEP57L1 | 3.46E-04 | 0.35097493 | 12 |
| SLC9A8 | 0 | 0.327394209 | 1 |
| SLC6A4 | 9.46E-06 | 0.352941176 | 3 |
| SLC6A3 | 4.56E-05 | 0.373728814 | 10 |
| CNOT6 | 1.83E-04 | 0.350138944 | 12 |
| MAPKAPK2 | 5.07E-04 | 0.390092879 | 18 |
| RGMB | 0 | 0.248310811 | 1 |
| SLC6A2 | 0 | 0.352658936 | 2 |
| KLC1 | 6.37E-04 | 0.40347667 | 16 |
| CEP44 | 0.001721281 | 0.361031519 | 10 |
| KIT | 0.001125443 | 0.396761134 | 47 |
| HBEGF | 8.68E-05 | 0.351674641 | 7 |
| MED4 | 0.004343681 | 0.406077348 | 33 |
| NOG | 0 | 0.248310811 | 3 |
| SLC2A5 | 0 | 0.28089172 | 1 |
| BMPER | 0 | 0.248310811 | 1 |
| SLC1A5 | 2.23E-04 | 0.405517241 | 8 |
| KIR2DL3 | 1.48E-05 | 0.369966443 | 4 |
| NOLC1 | 3.80E-04 | 0.397476341 | 20 |
| NDFIP1 | 6.04E-06 | 0.373728814 | 5 |
| KHK | 5.06E-05 | 0.343324251 | 7 |
| CD8A | 5.18E-06 | 0.342790517 | 6 |
| CD4 | 0.001931427 | 0.406826568 | 37 |
| CD247 | 8.00E-04 | 0.387181738 | 31 |
| CAMTA1 | 0 | 0.327394209 | 3 |
| KDR | 0.002173694 | 0.407202216 | 33 |
| CD5 | 1.29E-04 | 0.362962963 | 16 |
| CD2 | 5.72E-05 | 0.358828316 | 10 |
| CD3E | 6.15E-05 | 0.359559723 | 14 |
| KCNMA1 | 9.66E-05 | 0.362813657 | 5 |
| SRSF4 | 7.77E-05 | 0.385826772 | 12 |
| CCNB1 | 4.69E-04 | 0.386333771 | 26 |
| SEMA3F | 1.73E-04 | 0.333711691 | 4 |
| CBLB | 4.21E-04 | 0.414668547 | 30 |
| CBL | 0.011854431 | 0.461538462 | 108 |
| STXBP2 | 8.06E-06 | 0.325581395 | 2 |
| STXBP1 | 6.30E-04 | 0.402372263 | 19 |
| HGS | 0.003729055 | 0.436633663 | 41 |
| CAV1 | 0.007113273 | 0.462264151 | 63 |
| WFIKKN2 | 0 | 0.294983278 | 2 |
| JAK3 | 5.50E-04 | 0.381322957 | 23 |
| MED28 | 3.32E-04 | 0.380664653 | 16 |
| HIST2H3A | 9.72E-05 | 0.396048496 | 11 |
| JARID2 | 0 | 0.327394209 | 1 |
| AURKA | 0.005205847 | 0.442548921 | 58 |
| CNST | 1.11E-05 | 0.301332422 | 2 |
| PHC3 | 1.42E-04 | 0.335488779 | 2 |
| CNOT9 | 7.48E-04 | 0.383311604 | 15 |
| STAU1 | 0.002085811 | 0.437066402 | 54 |
| STAT3 | 0.006485129 | 0.446582278 | 73 |
| ITK | 3.26E-04 | 0.405330882 | 29 |
| STAT5A | 6.89E-04 | 0.415254237 | 34 |
| ITGA2 | 9.95E-04 | 0.367806505 | 12 |
| STAT1 | 0.004268276 | 0.432564983 | 56 |
| ST13 | 0.001018143 | 0.423427748 | 19 |
| IRF6 | 0 | 0.270635164 | 1 |
| CAMK2G | 4.02E-04 | 0.393398751 | 18 |
| CALR | 0.00237633 | 0.434268833 | 32 |
| IRF2 | 1.30E-04 | 0.381983543 | 10 |
| IRF1 | 9.51E-05 | 0.36371134 | 12 |
| CROT | 7.36E-05 | 0.333333333 | 2 |
| ADAM15 | 4.24E-05 | 0.375159507 | 10 |
| UHRF2 | 2.94E-04 | 0.379028792 | 19 |
| INSR | 0.003025564 | 0.427947598 | 50 |
| SSB | 5.59E-04 | 0.427740058 | 26 |
| TRIM21 | 6.41E-04 | 0.39392586 | 21 |
| MCM5 | 0.001178923 | 0.441883768 | 50 |
| SETX | 1.89E-04 | 0.373096447 | 8 |
| ADAMTS1 | 1.97E-04 | 0.316355811 | 4 |
| MCM2 | 0.01317039 | 0.470902296 | 110 |
| CA8 | 2.69E-04 | 0.357519254 | 6 |
| MBP | 3.14E-04 | 0.36857501 | 12 |
| ZNF292 | 7.01E-05 | 0.343591741 | 3 |
| MAPK8IP1 | 4.85E-04 | 0.402923709 | 14 |
| SDC4 | 6.67E-04 | 0.3528 | 10 |
| SDC1 | 5.53E-04 | 0.359266802 | 6 |
| MED20 | 6.05E-04 | 0.389232127 | 15 |
| MAPT | 0.002160055 | 0.445229682 | 48 |
| CNOT1 | 0.004013855 | 0.418803419 | 21 |
| MAP1B | 4.90E-04 | 0.415449835 | 23 |
| SNRNP200 | 0.001039865 | 0.437934459 | 41 |
| SH3BP5 | 1.53E-04 | 0.386164623 | 6 |
| EXOC6 | 2.28E-04 | 0.408144378 | 7 |
| C4B | 2.82E-04 | 0.374045802 | 7 |
| CCL5 | 0.015913212 | 0.390438247 | 38 |
| C4A | 2.82E-04 | 0.374045802 | 7 |
| PDGFC | 5.03E-05 | 0.310891787 | 3 |
| SERPING1 | 2.47E-04 | 0.355645161 | 5 |
| CCND1 | 7.64E-04 | 0.417613636 | 28 |
| BCL2L1 | 5.87E-04 | 0.39480752 | 21 |
| SMAD6 | 6.52E-04 | 0.359266802 | 16 |
| SCN5A | 4.15E-04 | 0.384816754 | 10 |
| SMAD2 | 0.004723492 | 0.439461883 | 60 |
| BAX | 6.11E-04 | 0.393398751 | 22 |
| SMAD3 | 0.007240639 | 0.458658346 | 62 |
| FAM189A2 | 1.17E-05 | 0.343190661 | 4 |
| BAD | 1.77E-04 | 0.404587156 | 16 |
| GRAP2 | 0.001571203 | 0.406264394 | 21 |
| LYN | 0.008910416 | 0.456049638 | 80 |
| LYZ | 0.005107759 | 0.409090909 | 37 |
| SH2D1A | 1.68E-05 | 0.378216123 | 13 |
| AXL | 1.84E-04 | 0.401456532 | 21 |
| TGFBRAP1 | 3.17E-04 | 0.336641221 | 7 |
| HTR3C | 1.07E-04 | 0.285252264 | 2 |
| ARHGAP15 | 2.46E-06 | 0.374363328 | 3 |
| GPHA2 | 0 | 0.24740533 | 1 |
| LTF | 1.13E-04 | 0.367653189 | 7 |
| LTC4S | 0 | 0.283601286 | 4 |
| NCBP2 | 5.86E-05 | 0.343591741 | 5 |
| CTSL | 7.24E-04 | 0.35708502 | 9 |
| LRP2 | 0.003237033 | 0.39269813 | 17 |
| LRP1 | 0.001936934 | 0.418604651 | 25 |
| LOC285074 | 0.001123696 | 0.362366475 | 10 |
| FZR1 | 7.01E-04 | 0.384648932 | 19 |
| CTSD | 0.002074435 | 0.404772832 | 18 |
| SORT1 | 0.001481871 | 0.384648932 | 18 |
| CTSB | 0.001374068 | 0.382314694 | 17 |
| S100A1 | 8.53E-05 | 0.358682391 | 10 |
| LPL | 0.007750109 | 0.384481255 | 26 |
| CTNND1 | 6.69E-04 | 0.434268833 | 25 |
| RYR2 | 2.40E-04 | 0.36 | 7 |
| LAMTOR4 | 2.03E-04 | 0.351674641 | 3 |
| CTNND2 | 3.13E-05 | 0.364613477 | 7 |
| PAG1 | 1.48E-04 | 0.369811321 | 17 |
| DDX19B | 0.002786591 | 0.396761134 | 22 |
| CTGF | 0.002195274 | 0.390092879 | 18 |
| CTLA4 | 1.32E-04 | 0.355788624 | 11 |
| ADAP2 | 2.08E-05 | 0.381322957 | 7 |
| EIF3G | 1.12E-04 | 0.402372263 | 11 |
| RREB1 | 2.65E-06 | 0.346289753 | 2 |
| DYNLL1 | 6.39E-04 | 0.413890192 | 28 |
| LIMS1 | 1.92E-04 | 0.357519254 | 10 |
| CST3 | 1.11E-04 | 0.340803709 | 8 |
| SOCS1 | 6.42E-04 | 0.407014305 | 30 |
| DOK3 | 5.87E-05 | 0.372151899 | 14 |
| VCAN | 7.48E-04 | 0.321077539 | 10 |
| CSNK2B | 0.006254508 | 0.433841613 | 41 |
| CHRD | 9.95E-04 | 0.349861166 | 3 |
| CSNK2A2 | 0.002005641 | 0.425470333 | 33 |
| CSNK2A1 | 1.73E-04 | 0.409470752 | 18 |
| CSNK1A1 | 4.82E-04 | 0.424650939 | 25 |
| CSNK1D | 3.91E-04 | 0.408333333 | 15 |
| CSK | 0.001828846 | 0.421606119 | 46 |
| CSF3R | 1.33E-04 | 0.380336352 | 16 |
| ILF3 | 9.07E-04 | 0.416234073 | 22 |
| RPS6KB2 | 0.002218942 | 0.432777233 | 46 |
| SKAP1 | 2.82E-04 | 0.390438247 | 13 |
| CSF2RB | 8.88E-05 | 0.372466216 | 20 |
| RIN3 | 3.21E-04 | 0.376923077 | 12 |
| PRSS3P2 | 0 | 0.327394209 | 1 |
| RLF | 0 | 0.327394209 | 1 |
| GRK1 | 3.61E-06 | 0.346834448 | 5 |
| TNFSF11 | 7.52E-04 | 0.376923077 | 15 |
| CRYAB | 0.00478989 | 0.437066402 | 29 |
| VPS35 | 3.97E-04 | 0.402372263 | 13 |
| CXCL8 | 6.22E-04 | 0.332830189 | 9 |
| DIP2A | 4.26E-04 | 0.317266187 | 5 |
| HAPLN1 | 2.67E-04 | 0.352941176 | 4 |
| CRK | 0.007741779 | 0.457231726 | 86 |
| IL2RB | 1.36E-04 | 0.388717497 | 20 |
| SIAH2 | 3.12E-04 | 0.384481255 | 14 |
| IKBKB | 0.002651306 | 0.421002387 | 48 |
| FAM71E2 | 0 | 0.327394209 | 1 |
| IRAK3 | 1.02E-04 | 0.347107438 | 6 |
| IGSF1 | 5.07E-04 | 0.304137931 | 9 |
| SIAH1 | 6.83E-04 | 0.396939694 | 21 |
| SHC1 | 0.006577478 | 0.457231726 | 96 |
| CHEK2 | 5.84E-04 | 0.390957447 | 17 |
| CLDN3 | 4.08E-04 | 0.355932203 | 4 |
| WFIKKN1 | 2.83E-04 | 0.322132944 | 3 |
| DCD | 2.92E-04 | 0.411380597 | 15 |
| SH3BP2 | 2.03E-04 | 0.387351779 | 18 |
| CBX4 | 5.43E-04 | 0.373412362 | 13 |
| IGHG2 | 1.32E-04 | 0.352095808 | 6 |
| IGHG1 | 0.001162731 | 0.410041841 | 18 |
| YBX3 | 4.89E-04 | 0.388546256 | 16 |
| HHIPL2 | 0 | 0.283510125 | 1 |
| FSTL1 | 7.41E-04 | 0.34751773 | 8 |
| IGFBP6 | 2.99E-05 | 0.299897994 | 3 |
| IGFBP5 | 7.73E-04 | 0.355645161 | 10 |
| IKBKG | 0.004539046 | 0.446808511 | 71 |
| IGFBP7 | 5.41E-04 | 0.324622746 | 5 |
| IGFBP2 | 0 | 0.286270691 | 2 |
| IGFBP1 | 0 | 0.286270691 | 2 |
| TMX2-CTNND1 | 6.69E-04 | 0.434268833 | 25 |
| LDB3 | 3.38E-05 | 0.350277998 | 3 |
| IGFBP4 | 1.22E-05 | 0.305190311 | 3 |
| IGFBP3 | 0.003336008 | 0.394630872 | 21 |
| RELA | 0.004725047 | 0.4375 | 59 |
| IGF1R | 0.001974337 | 0.439243028 | 37 |
| IGFALS | 2.21E-06 | 0.289655172 | 3 |
| REL | 0.001216426 | 0.405144695 | 28 |
| CISH | 7.51E-05 | 0.375478927 | 12 |
| IGF1 | 0.004744725 | 0.342524272 | 18 |
| SLC39A4 | 2.00E-05 | 0.317266187 | 3 |
| APOA5 | 1.66E-05 | 0.302884615 | 2 |
| RBP4 | 6.33E-05 | 0.371368421 | 4 |
| IFNAR1 | 3.51E-05 | 0.365975104 | 12 |
| IFNA14 | 0 | 0.278409091 | 1 |
| FGFR1OP | 5.78E-04 | 0.363411619 | 15 |
| RASA1 | 0.002796232 | 0.44321608 | 54 |
| CHKB | 0 | 0.327394209 | 1 |
| UNC119 | 4.51E-04 | 0.38381201 | 16 |
| IDE | 4.29E-04 | 0.39516129 | 9 |
| RANGAP1 | 9.41E-04 | 0.388717497 | 15 |
| RANBP2 | 0.001390797 | 0.406826568 | 23 |
| WDR5 | 0.002620777 | 0.414863594 | 21 |
| RALGDS | 3.49E-05 | 0.365217391 | 7 |
| TPPP | 3.07E-05 | 0.380172414 | 7 |
| RAF1 | 0.001882018 | 0.434696895 | 53 |
| TGS1 | 0 | 0.336769759 | 2 |
| RAB5C | 0.002005809 | 0.414084507 | 30 |
| CETP | 1.70E-05 | 0.299287411 | 4 |
| CEP63 | 2.95E-04 | 0.345070423 | 10 |
| GPRC5A | 3.27E-04 | 0.34765471 | 17 |
| SH2D2A | 8.67E-05 | 0.386164623 | 19 |
| RAB13 | 5.33E-05 | 0.299287411 | 3 |
| RAB5A | 0.002848318 | 0.399456522 | 26 |
| RAB3A | 1.40E-04 | 0.390957447 | 10 |
| ITIH1 | 2.54E-04 | 0.365671642 | 4 |
| SOCS3 | 8.17E-04 | 0.415645617 | 31 |
| ITGB5 | 0.001224965 | 0.382149047 | 15 |
| PXN | 0.004336196 | 0.432564983 | 59 |
