# Supplementary material for: Exploring the Potential Mechanism of Tang-Shen-Ning Decoction against Diabetic Nephropathy Based on the Combination of Network Pharmacology and Experimental Validation
Source: Evid Based Complement Alternat Med. 2021 Sep 9;2021:1025053. doi: 10.1155/2021/1025053 (PMC8445713; doi:10.1155/2021/1025053)
Supplement: Supplementary Materials. — Supplementary material 1: Table S1. The details of DN-DEGs in GSE30528. Supplementary material 2: Table S2. The information about topological parameters in the PPI network. Supplementary material 3: Table S3. The information of GFR and TSN-related DN-DEGs provided by Nephroseq v5. Supplementary material 4: Figure S1. Gene relative expression of TSN-related DN-DEGs in GSE30528. (A) 9 upregulated targets; (B) 14 downregulated targets. Supplementary material 5: Figure S2. Correlation between TSN-related DN-DEGs expression and GFR. [file 1025053.f1.zip › 1025053.f1/Table S3.doc]

| **Data of Gene expression and GFR in GSE30528** | | | | | | |
| --- | --- | --- | --- | --- | --- | --- |
| **Sample Name** | 100_control | 110_control | 119_control | 123_control | 150_control | 151_DN |
| **Log2 GFR (MDRD) (ml/min/1.73m2)** | 6.106078 | 6.507795 | 5.930738 | 6.108524 | 5.78136 | 4.523562 |
| **Median-centered Log2 MMP7 Expression** | 2.31023 | 2.29623 | 2.29882 | 1.07428 | 1.64663 | 4.3723 |
| **Median-centered Log2 ADH1B Expression Value** | -0.47948 | 0.67328 | -0.09821 | -0.59614 | -0.47727 | 1.85077 |
| **Median-centered Log2 MOXD1 Expression Value** | 0.14221 | -0.20933 | 0.06415 | -1.07118 | -0.68436 | 0.33911 |
| **Median-centered Log2 CCL5 Expression Value** | -1.39124 | -0.63488 | 1.40773 | -1.02208 | -1.01806 | -0.73675 |
| **Median-centered Log2 AKR1B10 Expression Value** | -0.95953 | -1.21434 | -1.37487 | -1.34881 | -1.09916 | 0.93105 |
| **Median-centered Log2 LCK Expression Value** | -1.17525 | -0.86745 | -0.26933 | -1.33342 | -1.35366 | -0.43532 |
| **Median-centered Log2 ALOX5 Expression Value** | -1.64078 | -1.54724 | -1.64937 | -1.76381 | -1.67675 | -1.58171 |
| **Median-centered Log2 IRF8 Expression Value** | 2.14247 | 2.32727 | 1.43653 | 0.77619 | 1.29259 | 2.08158 |
| **Median-centered Log2 VEGFA Expression Value** | 2.41269 | 3.34057 | 3.71396 | 2.92758 | 3.24903 | 1.07842 |
| **Median-centered Log2 GADD45B Expression Value** | 4.83645 | 4.93878 | 4.89906 | 5.47999 | 5.13198 | 2.60677 |
| **Median-centered Log2 CLDN5 Expression Value** | 3.63294 | 4.25545 | 5.2125 | 4.9974 | 4.45568 | 2.01112 |
| **Median-centered Log2 ALB Expression Value** | 2.88834 | 0.95093 | -1.9048 | 2.8314 | -0.47847 | -1.67366 |
| **Median-centered Log2 BMP2 Expression Value** | 3.57804 | 4.28863 | 4.30571 | 2.37402 | 3.33745 | 0.11971 |
| **Median-centered Log2 GJA1 Expression Value** | 5.39144 | 4.71879 | 5.35165 | 4.63489 | 4.60742 | 2.0484 |
| **Median-centered Log2 CTGF Expression Value** | 5.9292 | 5.19671 | 6.77768 | 5.25941 | 6.30133 | 1.5944 |
| **Median-centered Log2 HPGD Expression Value** | 5.75889 | 5.26881 | 5.24665 | 4.61058 | 5.45905 | 3.78016 |
| **Median-centered Log2 F3 Expression Value** | 4.56653 | 3.9046 | 4.55158 | 4.45018 | 3.69371 | 0.28894 |
| **Median-centered Log2 SNCA Expression Value** | 4.83535 | 3.0537 | 3.81273 | 3.5409 | 4.3653 | -0.57001 |
| **Median-centered Log2 PLAT Expression Value** | 3.64989 | 3.10033 | 6.77115 | 2.40064 | 3.66966 | 1.15538 |
| **Median-centered Log2 GPRC5A Expression Value** | 3.44875 | 3.4204 | 3.78223 | 3.74883 | 4.33053 | 0.10967 |
| **Median-centered Log2 IGF1 Expression Value** | 4.25446 | 2.9305 | 2.19335 | 3.80618 | 4.04446 | -0.69763 |
| **Median-centered Log2 LPL Expression Value** | 5.2476 | 3.5421 | 3.33392 | 3.57681 | 4.97413 | -1.19398 |

(Continued Table)

| **Data of Gene expression and GFR in GSE30528** | | | | | | |
| --- | --- | --- | --- | --- | --- | --- |
| **Sample Name** | 154_control | 158_DN | 164_DN | 168_DN | 178_DN | 33_control |
| **Log2 GFR (MDRD) (ml/min/1.73m2)** | 6.044394 | 5.169925 | 4 | 5.246078 | 4.169925 | 6.70044 |
| **Median-centered Log2 MMP7 Expression** | 2.3169 | 0.6511 | 4.6091 | 5.11582 | 4.23698 | 2.14939 |
| **Median-centered Log2 ADH1B Expression Value** | -0.50428 | 1.19234 | 2.97903 | 3.26306 | 1.98774 | -0.11335 |
| **Median-centered Log2 MOXD1 Expression Value** | -0.37192 | 0.75813 | 2.5477 | 2.82956 | 1.13294 | -0.35277 |
| **Median-centered Log2 CCL5 Expression Value** | -1.27491 | -1.57427 | 1.29287 | 1.05421 | 0.75186 | -1.26737 |
| **Median-centered Log2 AKR1B10 Expression Value** | -0.23633 | 2.52617 | 4.32716 | -0.86007 | 3.22934 | -0.24689 |
| **Median-centered Log2 LCK Expression Value** | -1.37216 | -0.75362 | 0.89605 | 0.85247 | -0.35777 | -1.61069 |
| **Median-centered Log2 ALOX5 Expression Value** | -1.46764 | -1.56326 | -0.53805 | -1.35515 | -1.62849 | -1.95661 |
| **Median-centered Log2 IRF8 Expression Value** | 0.6449 | 1.33851 | 3.36532 | 3.9767 | 1.80921 | 1.3979 |
| **Median-centered Log2 VEGFA Expression Value** | 3.0206 | 2.07767 | 1.00752 | 1.48259 | 2.46394 | 3.52804 |
| **Median-centered Log2 GADD45B Expression Value** | 5.35894 | 4.48283 | 2.8024 | 4.36475 | 4.65034 | 4.94719 |
| **Median-centered Log2 CLDN5 Expression Value** | 4.63202 | 3.62162 | 1.34378 | 2.71194 | 4.02166 | 5.18958 |
| **Median-centered Log2 ALB Expression Value** | 1.49979 | -0.07394 | -1.35193 | 0.60383 | -1.94038 | -1.01446 |
| **Median-centered Log2 BMP2 Expression Value** | 2.67514 | 0.72858 | 1.53769 | 2.53795 | 1.06418 | 2.07154 |
| **Median-centered Log2 GJA1 Expression Value** | 4.63045 | 3.43705 | 3.37225 | 3.36279 | 2.12314 | 4.8148 |
| **Median-centered Log2 CTGF Expression Value** | 6.05622 | 4.16119 | 4.49856 | 5.37607 | 4.38089 | 5.3052 |
| **Median-centered Log2 HPGD Expression Value** | 6.0779 | 4.87231 | 4.13293 | 4.29561 | 4.13048 | 5.20873 |
| **Median-centered Log2 F3 Expression Value** | 4.1492 | 2.82552 | 1.27236 | 2.64442 | 3.22087 | 4.34445 |
| **Median-centered Log2 SNCA Expression Value** | 4.18938 | 1.11488 | 0.45957 | 1.08652 | 2.80326 | 3.77526 |
| **Median-centered Log2 PLAT Expression Value** | 2.66558 | 2.2991 | 2.06137 | 2.08024 | 4.87922 | 4.3491 |
| **Median-centered Log2 GPRC5A Expression Value** | 3.2781 | 2.13821 | 0.51623 | 1.37221 | 2.45083 | 4.63154 |
| **Median-centered Log2 IGF1 Expression Value** | 4.44933 | -0.70691 | -0.26198 | 0.62508 | 0.74139 | 3.44089 |
| **Median-centered Log2 LPL Expression Value** | 4.4711 | 3.2647 | 0.68572 | 1.3385 | 2.61271 | 5.38081 |

(Continued Table)

| **Data of Gene expression and GFR in GSE30528** | | | | | | |
| --- | --- | --- | --- | --- | --- | --- |
| **Sample Name** | 34_control | 40_DN | 48_DN | 62_DN | 67_DN | 76_control |
| **Log2 GFR (MDRD) (ml/min/1.73m2)** | 6.857981 | 4.70044 | 5.857981 | 4.584962 | 5.349903 | 6.768184 |
| **Median-centered Log2 MMP7 Expression** | 2.42987 | 4.40757 | 1.174 | 5.04159 | 4.7932 | 2.17411 |
| **Median-centered Log2 ADH1B Expression Value** | 0.29058 | 4.18302 | -0.75884 | 2.52399 | 0.94954 | 0.32239 |
| **Median-centered Log2 MOXD1 Expression Value** | 0.20618 | 2.75418 | -0.30717 | 3.28425 | 1.49408 | -0.01556 |
| **Median-centered Log2 CCL5 Expression Value** | -0.60836 | 1.74203 | -0.87458 | 1.64367 | 1.33378 | -1.43749 |
| **Median-centered Log2 AKR1B10 Expression Value** | -1.25446 | 0.09627 | -0.52608 | -1.24121 | -1.45239 | -1.33032 |
| **Median-centered Log2 LCK Expression Value** | -1.39709 | 0.50569 | -0.47202 | 0.91741 | 2.11934 | -1.42523 |
| **Median-centered Log2 ALOX5 Expression Value** | -1.92208 | -1.90819 | -1.54271 | -1.49093 | -0.93438 | -1.76177 |
| **Median-centered Log2 IRF8 Expression Value** | 1.0897 | 3.72688 | 2.26185 | 3.20903 | 4.43525 | 1.29492 |
| **Median-centered Log2 VEGFA Expression Value** | 2.37957 | 1.27133 | 2.52668 | 0.41971 | 1.78059 | 3.30702 |
| **Median-centered Log2 GADD45B Expression Value** | 5.78102 | 3.9253 | 4.83282 | 2.60048 | 3.34259 | 6.20282 |
| **Median-centered Log2 CLDN5 Expression Value** | 4.31889 | 2.34147 | 3.72545 | 1.73335 | 2.80797 | 4.70959 |
| **Median-centered Log2 ALB Expression Value** | -1.90303 | -2.37418 | -2.08772 | -2.10595 | -0.03018 | -1.46922 |
| **Median-centered Log2 BMP2 Expression Value** | 2.03659 | 2.13572 | 2.79789 | 0.78942 | 1.14135 | 4.33345 |
| **Median-centered Log2 GJA1 Expression Value** | 5.34901 | 3.74761 | 4.49403 | 2.95732 | 2.94637 | 5.56895 |
| **Median-centered Log2 CTGF Expression Value** | 5.68083 | 3.1464 | 4.01288 | 2.74792 | 4.79927 | 6.92578 |
| **Median-centered Log2 HPGD Expression Value** | 4.7048 | 3.40546 | 5.24196 | 1.36174 | 4.12158 | 6.80445 |
| **Median-centered Log2 F3 Expression Value** | 3.9686 | 0.99087 | 3.70088 | 0.41707 | 2.59821 | 4.89328 |
| **Median-centered Log2 SNCA Expression Value** | 3.14391 | 0.6196 | 2.03188 | 0.84721 | 1.34364 | 3.7276 |
| **Median-centered Log2 PLAT Expression Value** | 4.19985 | 1.4006 | 1.97416 | 0.17512 | 3.1668 | 4.55503 |
| **Median-centered Log2 GPRC5A Expression Value** | 4.15038 | 1.58392 | 2.92659 | 1.3111 | 2.96654 | 4.86635 |
| **Median-centered Log2 IGF1 Expression Value** | 0.2628 | 1.29421 | -0.26652 | 0.95553 | 0.91837 | 2.8451 |
| **Median-centered Log2 LPL Expression Value** | 4.29433 | 0.65728 | 2.7544 | -0.72488 | 2.6741 | 5.27825 |

(Continued Table)

| **Data of Gene expression and GFR in GSE30528** | | | | |
| --- | --- | --- | --- | --- |
| **Sample Name** | 77_control | 81_control | 82_control | 83_control |
| **Log2 GFR (MDRD) (ml/min/1.73m2)** | 6.70044 | 6.643856 | 5.807355 | 5.70044 |
| **Median-centered Log2 MMP7 Expression** | 0.24055 | 1.08263 | 2.15286 | 1.11294 |
| **Median-centered Log2 ADH1B Expression Value** | -1.28867 | 0.06924 | 0.68919 | 0.17132 |
| **Median-centered Log2 MOXD1 Expression Value** | -0.30231 | -0.42853 | -0.26382 | 0.10272 |
| **Median-centered Log2 CCL5 Expression Value** | -1.48351 | -1.39146 | -1.40033 | -1.54908 |
| **Median-centered Log2 AKR1B10 Expression Value** | 0.13665 | -1.20367 | -0.91481 | -1.07355 |
| **Median-centered Log2 LCK Expression Value** | -1.17827 | -1.72283 | -1.56056 | -1.55906 |
| **Median-centered Log2 ALOX5 Expression Value** | -1.88645 | -1.75334 | -1.83923 | -1.58859 |
| **Median-centered Log2 IRF8 Expression Value** | 1.11706 | 2.08757 | 2.73878 | 0.58197 |
| **Median-centered Log2 VEGFA Expression Value** | 3.05602 | 3.49549 | 2.77712 | 3.03332 |
| **Median-centered Log2 GADD45B Expression Value** | 5.63408 | 5.53072 | 7.01782 | 4.65901 |
| **Median-centered Log2 CLDN5 Expression Value** | 4.08825 | 4.96852 | 4.42682 | 4.54347 |
| **Median-centered Log2 ALB Expression Value** | 2.53467 | 2.83796 | 0.94131 | 0.19865 |
| **Median-centered Log2 BMP2 Expression Value** | 3.00817 | 2.90074 | 4.1288 | 3.4306 |
| **Median-centered Log2 GJA1 Expression Value** | 5.71547 | 5.78395 | 5.53182 | 5.40363 |
| **Median-centered Log2 CTGF Expression Value** | 6.03225 | 6.9936 | 7.06316 | 5.76223 |
| **Median-centered Log2 HPGD Expression Value** | 6.60647 | 6.03546 | 5.46663 | 5.90409 |
| **Median-centered Log2 F3 Expression Value** | 4.4949 | 4.9539 | 4.36577 | 4.7827 |
| **Median-centered Log2 SNCA Expression Value** | 3.86032 | 3.86264 | 2.59878 | 1.75596 |
| **Median-centered Log2 PLAT Expression Value** | 5.0275 | 5.35663 | 5.14199 | 5.29974 |
| **Median-centered Log2 GPRC5A Expression Value** | 4.31784 | 4.27619 | 4.85657 | 4.2242 |
| **Median-centered Log2 IGF1 Expression Value** | 0.18306 | 0.32337 | 3.0418 | 3.87201 |
| **Median-centered Log2 LPL Expression Value** | 4.79637 | 4.63051 | 4.67069 | 5.5894 |
